# A global phylogenomic and metabolic reconstruction of the large intestine bacterial community of domesticated cattle

**DOI:** 10.1186/s40168-022-01357-1

**Published:** 2022-09-26

**Authors:** S. Teseo, S. Otani, C. Brinch, S. Leroy, P. Ruiz, M. Desvaux, E. Forano, F. M. Aarestrup, P. Sapountzis

**Affiliations:** 1 School of Biological Sciences, Nanyang Technological University, Singapore, Singapore; 2 National Food Institute, Technical University of Denmark, Kongens Lyngby, Denmark; 3 Université Clermont Auvergne, INRAE, UMR 0454 MEDIS, Clermont-Ferrand, France

## Abstract

**Background:**

The large intestine is a colonization site of beneficial microbes complementing the nutrition of cattle but also of zoonotic and animal pathogens. Here, we present the first global gene catalog of cattle fecal microbiomes, a proxy of the large intestine microbiomes, from 436 metagenomes from six countries.

**Results:**

Phylogenomics suggested that the reconstructed genomes and their close relatives form distinct branches and produced clustering patterns that were reminiscent of the metagenomics sample origin. Bacterial taxa had distinct metabolic profiles, and complete metabolic pathways were mainly linked to carbohydrates and amino acids metabolism. Dietary changes affected the community composition, diversity, and potential virulence. However, predicted enzymes, which were part of complete metabolic pathways, remained present, albeit encoded by different microbes.

**Conclusions:**

Our findings provide a global insight into the phylogenetic relationships and the metabolic potential of a rich yet understudied bacterial community and suggest that it provides valuable services to the host. However, we tentatively infer that members of that community are not irreplaceable, because similar to previous findings, symbionts of complex bacterial communities of mammals are expendable if there are substitutes that can perform the same task.

Video Abstract

**Supplementary Information:**

The online version contains supplementary material available at 10.1186/s40168-022-01357-1.

## Introduction

Microbial processing of plant material in the rumen, the main fermentation compartment in the foregut, enables ruminants to exploit plant biomass nutrients inaccessible to monogastric animals. This allowed them to conquer an untapped ecological niche around 20–30 mya [1] and then, starting from the Neolithic, led to a series of domestication events, making them irreplaceable associates of humans [2]. Relying exclusively on plant material, domesticated cattle supply dairy products, meat, and leather and have physically contributed to agriculture (i.e., as a means of transport or via traction of work machinery). In addition, today’s ever-growing cattle population provides essential services to agriculture, for example, grazing unusable land to exploitability or recycling cellulose-rich by-products of industrial processes (e.g., distillers grains) [3, 4]. However, it comes with a cost as ruminants are also major contributors to methane greenhouse gas emissions [5]. Genomic approaches have uncovered the rumen microbe functions, improving our knowledge of resident communities and their metabolic relationships with animal hosts, as well as among coexisting strains, and identifying most of the functional diversity [6–10].

While rumen microorganisms serve as necessary mediators in cattle nutrition, the microbial contribution to cattle digestion continues in the small and large intestine. There, water and nutrients continue to be absorbed [11, 12], and resident microbes break down partially digested feed [13, 14]. With a microbial abundance second only to the rumen itself, the large intestine has critical relevance for bovine production. This depends not only on the effects of the metabolic processes of its resident bacterial community but also — and especially in juvenile individuals — on the presence of pathogens, which can harm both animals and human consumers. The most common pathogens colonizing the large intestine include Shiga-toxigenic *Escherichia coli* (STEC) strains, other *Escherichia* and *Salmonella* pathotypes colonizing the gastrointestinal tract (GIT) via the fecal-oral route [15, 16], and *Clostridium perfringens* [17], *Campylobacter* [18], and *Listeria monocytogenes* [19] strains. Determining microbial activities occurring in the large intestine of cattle is therefore key to meat and dairy safety [20] as well as for the welfare of animals, especially in early life [21].

Surprisingly, studies on the microbial communities of bovine large intestine rarely go beyond the characterization of community composition — mostly via 16S/18S rRNA metabarcoding [13] — or only characterize single bacterial species of interest such as those threatening animals or human consumers [15, 22–24]. While these approaches have undoubtedly advanced our knowledge in the field, only functional metagenomic investigations can elucidate potential links between phylogenetic diversity and genomic inventory and make predictions about the functional roles of bacterial taxa. Even though studies have applied this approach to the cattle’s large intestine, they have exclusively focused on either identifying antimicrobial resistance genes [25–34] or comparing gene catalogs across different gut compartments [35]. This renders the knowledge of the roles that bacteria play in the cattle intestine still fragmentary.

Here, we have used shotgun metagenomics data to perform a thorough characterization of the bacterial communities of cattle feces. Relying on metagenomes from 436 samples collected across six countries from ten previous studies [25–34], 70 of which are newly sequenced in this study, we have reconstructed a representative set of metagenome-assembled genomes (MAGs), using stringent criteria to make them maximally representative of bacterial genomes from cattle feces. To examine how such genomes (represented by our MAGs) may have diverged from bacterial symbionts of the rumen or other close relatives and examine their origins, we reconstructed phylogenies using single-copy orthologs for each class, which we used to compare MAGs to each other and non-fecal-related genomes. We estimated the MAGs’ abundances and functionally characterized and compared bacterial genomic inventories in the context of two common dietary transitions ordinarily taking place in domesticated cattle: the introduction of solid feed in young calves and the annual rotation between winter and summer diets in dairy cows. All comparisons were performed using the dereplicated reconstructed MAG dataset and a customized (relevant for cattle) virulence database to assess potential pathogenic threats, either general or related to these specific dietary changes. Our results suggest that the cattle host likely benefits from the microbial metabolism in the large intestine, even though dietary shifts correlate to massive changes not only in microbial diversity and richness but also in metabolic potential. Regardless, the pathways related to the fermentation of sugars, the production of short-chain fatty acids (SCFAs), and the metabolism of amino acid (AA) to produce lysine, threonine — two of the three limiting AAs in cattle feed [36, 37] — and arginine remain present through various predicted enzymes that are likely encoded by different taxa.

## Results

### Metagenome sequencing and the core microbiome of the cattle fecal microbiome

After trimming and removing bovine host reads, the 70 fecal samples of dairy Holstein cows collected for this study, along with 366 additional metagenomic samples produced in ten previous studies (Table S1), yielded approximately 2.6 terabytes of sequencing reads, which were individually assembled (Table S2). The taxonomic classification of the predicted open reading frames (ORFs) from the contigs showed that > 61% of the reads originated from bacteria, ca 0.45% from archaea, 0.14% from Eukaryota, and 0.26% from viruses. A little less than 38% of readings were unclassified (Table S3). In this study, we only focused on bacterial contigs, the majority of which appeared to originate from Bacteroidia and Clostridia.

**Fig. 1:**
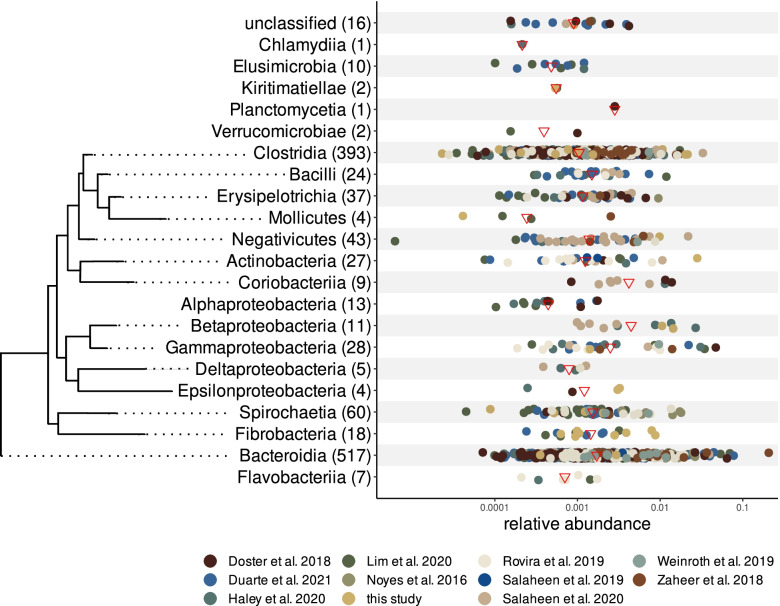
Overview of the dereplicated set of 1232 MAGs. On the left, a summary of the phylogenetic relationships of MAGs in our study. The tree is based on a universal ML tree built using the best quality MAGs (Sup. Res. 1; “common tree”). Between parentheses, the total number of MAGs for each class. Scatterplot shows the mean proportional abundance (see “Methods” & Sup. Res. 1) of each MAG in all 436 samples (color coded) and the overall mean for each class (red triangles)

The individual metagenomic binning of each assembly produced a total of 34,600 metagenome-assembled genomes (MAGs). We retained 2114 of these after removing those of less than 85% completeness and more than 5% contamination (based on a set of single-copy genes; checkM; [38]). The filtered subset belonged to 19 taxonomic classes and 13 phyla (Table S4A) and mostly fell within the Bacteroidetes (*n* = 848, > 40%) and Firmicutes (*n* = 795, > 37%) groups, followed by Actinobacteria (*n* = 149, > 7%), a partitioning somewhat reminiscent of that recently shown for the rumen [10]. Dereplication revealed 1232 unique MAGs (based on a 99% nucleotide similarity of their contigs; Fig. 1), which we used as reference sequences for most downstream analyses — we will henceforth refer to them as core MAGs. We used these as a reference template upon which we mapped the sequencing reads from our 436 samples, showing that on average, 27% of the reads (10–50%) could be mapped to them (Table S4B). Considering that we successfully assembled to contigs ~65% of the reads, and approximately 40% of all reads originated from bacteria, we estimate that the core MAGs covered on average 69.5% of the bacterial contigs assembled in our study (Table S4B).

### Phylogenomics

We examined the phylogenetic relationships of the 2114 high-quality MAGs separately for each class. These class-specific alignments composed of single-copy, non-recombinant orthologs (25-128 genes) phylogenies, were also used for downstream analyses. We used a total of 74 Hungate 1000 genomes [8] and 102 NCBI complete genomes (RefSeq) for the phylogenomic reconstructions after their predicted proteins gave clear matches to the 2114 MAGs amino acid (AA) sequences (Table S4A). The maximum likelihood AA phylogenetic trees showed that some fecal MAGs formed separate branches, and this was most evident in the Mollicutes, Fibrobacteria, Negativicutes, *α*- and *β*-Proteobacteria phylogenies (Sup. Res. 1). MAGs in the same branch often originated from samples of the same study (Sup. Res. 1), and these patterns were very clear for Spirochaetia, Bacteroidia, and Fibrobacteria. Most classes contained MAGs from all countries (and all studies) — especially the numerous Bacteroidia and Clostridia — but Coriobacteriia MAGs originated only from samples collected in the USA, Fibrobacteres only from samples from the EU and Korea, and *δ*-Proteobacteria only from samples from France, the Netherlands, and the USA.

For each reconstructed phylogeny, we formally examined the correlation between phylogenetic distances and the MAG origin (i.e., study ID which includes confounding effects of differences in location, animal, diet, farm, etc.) as well as the sample type (i.e., pen floor, composite, or individual in a separate test), and these comparisons confirmed some of our visual inferences (Sup. Res. 1; Table S5). Bacilli, Clostridia, Bacteroidia, *β*- and *δ*-Proteobacteria, Fibrobacteria, and Spirochaetia phylogenetic distances correlated significantly to differences explained by the study ID the MAGs originated from (*fdr* < 0.01) and Bacteroidia, Fibrobacteria, and Spirochaetia to phylogenetic distances to the sample type (*fdr* < 0.004).

### Functional annotation and the emergence of specific genes in MAGs associated with cattle

We functionally annotated the dereplicated set of 1232 MAGs to characterize their predicted open reading frames (ORF) on the basis of their AA similarities with the eggNOG database that included KEGG, GO, and EC annotations [39]. Functional annotation revealed a diverse repertoire of bacterial genes likely originating from facultative to strict anaerobes, as the number of enzymes related to oxidative phosphorylation varied greatly across MAGs. For example, the MAG *Psychrobacter_like_1* included a complete NADH respiratory chain as well as cytochrome c-related genes, suggesting that taxa similar to *Psychrobacter* represented bacterial members either capable of living outside the GIT environment or originating from the farm environment (i.e., the pen floor).

We mapped the complementary contributions of the 1232 core MAGs to more than ten thousand predicted enzyme sequences related to a series of processes including nutrient uptake, fermentation, biosynthesis, and housekeeping functions. To simplify the metabolic pathway data and identify the ones that are more likely important for the large intestine, we focused on the 210 samples originating from individual animal feces (Table S1), since these were more likely to have sequences originating from the large intestine bacterial community compared to pen floor and composite samples. We filtered the MAG contigs by selecting only those that were present in at least 80% of the 210 individual samples and were among the top 1000 most abundant bacterial contigs. This revealed 622 common contigs that contained > 2.5 k ORFs and corresponded to 1134 unique KEGG orthologs (KOs) coming from 114 MAGs (Table S6). We mapped these KOs to 175 relevant metabolic pathways (after excluding human specific, other nonbacterial and irrelevant pathways; see “Methods” and Table S6). The predicted enzymes mapped successfully multiple times to pathways related to the uptake of mono- and oligosaccharides, AAs and other essential nutrients (ABC transporters), sugar fermentation and AA catabolism (primarily), vitamin biosynthesis (e.g., cobalamin and thiamine), and several essential reactions involved in growth and survival that we will henceforth refer to as housekeeping (Table S6). We then focused only on enzymes that belonged to pathways that were complete in at least some of the 1232 MAGs (Sup. Res 2A). Apart from the housekeeping (cell wall, ribosome, etc.), the most common pathways were related to anaerobic fermentation of mono- and oligosaccharides, the catabolism of aspartate to produce lysine, threonine, and ectoine, and the production of arginine (Table S6C; Table S9 — lysine; Sup. Res. 2A).

**Fig. 2:**
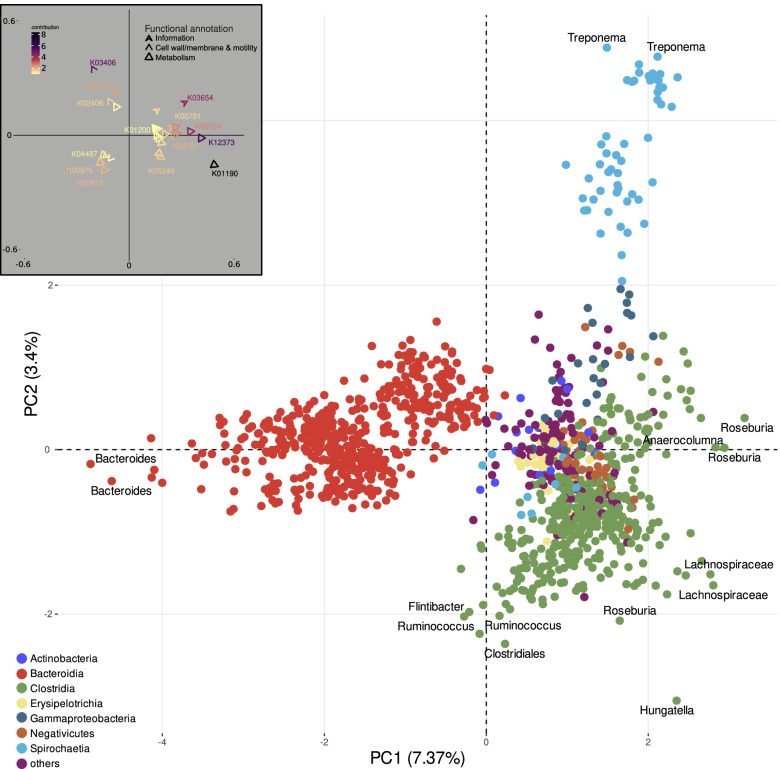
Functional separation of MAGs according to the eggNOG annotation of their ORFs. MAGs are color coded based on their phyla (identified by comparison with the NR database). The bacterial classes of MAGs towards the edges of the PCA plot are spelled out with the same color font. The most influential features (top 25) responsible for the separation of the MAGs are presented on the small panel at the top left. Features (vectors) are presented as different types of triangles to illustrate the eggNOG category to which they were assigned and are color coded based on the contribution of each feature. Their transparency is negatively associated with the quality of representation

We took advantage of our class-specific phylogenies to examine the potential emergence of enzymes as a result of the MAGs’ association with the fecal microbiome. Using the KOs annotation and a parsimony analysis, we examined each class’ phylogenetic tree and inferred functional KOs that may have evolved along the tree branches. Instead of comparing the MAGs on the basis of their taxonomic classification, this method took into account the phylogenetic reconstruction within each class to infer features (KOs) that may have putatively emerged. Considering that we compared MAGs to (almost or entirely) complete Hungate 1000 and RefSeq genomes, we focused on gains and expansions that took place multiple times and exclusively in the MAGs (Table S7A). While we found many gains and expansions occurred only once (i.e., as an isolated incident in a single class), a threonine aldolase and several predicted enzymes related to transporters and stress response recurred across multiple classes of fecal MAGs (Table S7A). When examining differences in the functional profiles among classes (on the basis of their taxonomic classification and functional annotation via the KOs of their predicted ORFs), ordination clearly differentiated Bacteroidia, Clostridia, and Spirochaetia (Fig. 2), and the most influential ORFs differentiating them were related to metabolism (Table S7B; Fig. 2).

### Microbiome changes as a result of dietary shifts

**Fig. 3:**
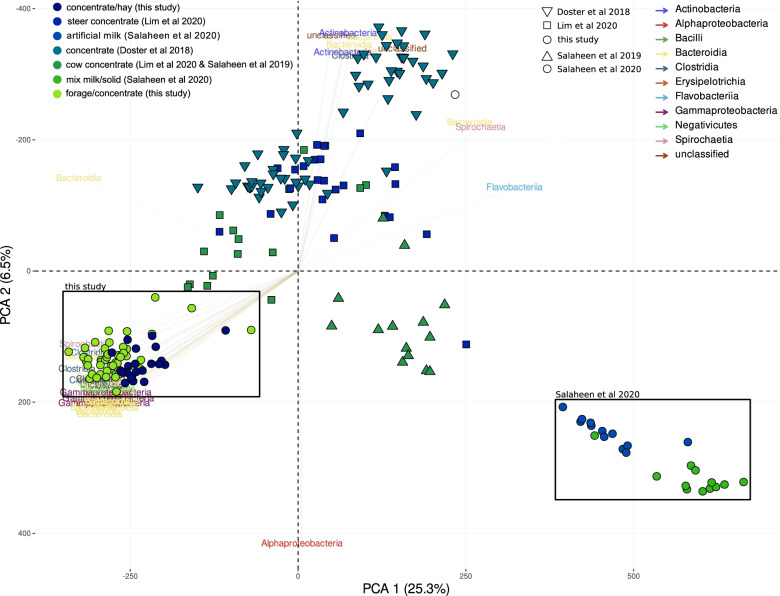
Beta diversity of the core MAGs in 208 feces samples of individual cattle, for which we had information about diet and collection time. The ordination is based on a principal coordinate analysis (PCoA) of Euclidean distances among the CLR-transformed values of the MAG compositional matrix. Samples are distinguished by diet (color coding) and study (shapes). The two subsets used for formal statistical comparisons are presented with filled circles and highlighted by black squares and light gray background. Vectors show the top 100 most influential features driving the differences and are color coded (bacterial class). Vector transparency is negatively related to the contribution of vectors

Principal coordinate ordination using the 1232 core MAGs and the samples from individual animals suggested that study ID drove the principal differences among samples (Fig. 3). We therefore set out to perform formal statistical comparisons only between groups of samples originating from the same study.

First, we examined the 70 samples sequenced for this study with a common origin (Theix, France) and matching collection, animals, DNA extraction, library preparation, and sequencing conditions. This showed that when we used the MAG compositional differences, the fecal communities of cows that were allowed to graze while having ab libitum access to a summer diet compared to those collected from cows fed a winter diet and were enclosed in pens differed significantly (PERMANOVA *p* = 0.001), even though this explained only a small proportion of the microbiome variation (*R2* = 0.075). The host had an equally significant effect (*p* = 0.001) and explained most of the variation (*R2* = 0.7), while alpha diversity and richness decreased from summer to winter (Kruskal-Wallis Sobs = 12.7, *p* < 0.001; Kruskal-Wallis Shannon = 23.6, *p* < 0.001). A differential representation analysis of the MAG contigs also highlighted the higher richness of samples coming from cows fed the summer diet (allowed to graze), as more than 81% of the total ORFs identified within the differentially represented (DifRep) contigs were specific to summer samples. When we looked at features specific to summer samples, 50% of the contigs came from only 21 MAGs (Table S8). Within these 21 summer-specific MAGs, > 25% of their predicted ORFs were specific to summer sample sequences (median: 41%, range: 29–68%). Contrarily, when looking at winter-specific MAGs, only *Bifidobacterium_angulatum_like_2* had > 25% of their ORFs DifRep, while only five had > 10% of their predicted ORFs exclusively present in winter samples (Table S8).

**Fig. 4:**
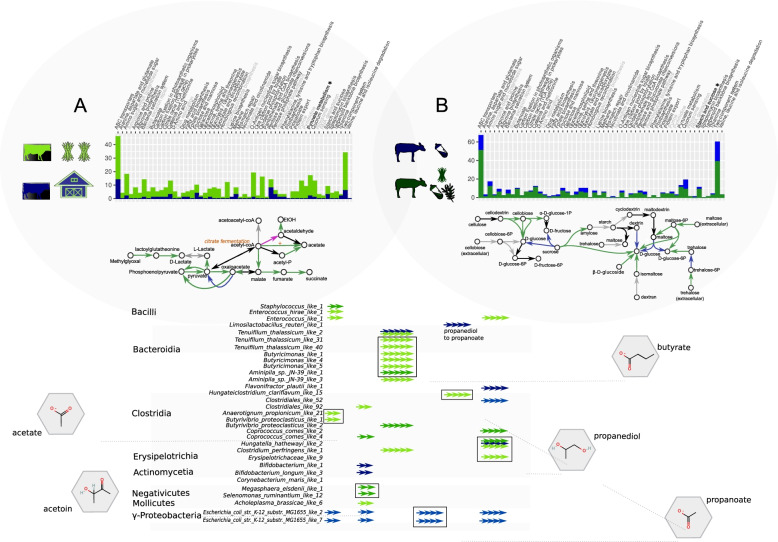
Potential metabolic changes **A** in Holstein dairy cows that have shifted from summer to winter diets and **B** in calves before and after introduction of solid feed (**B**). The barplots show the number of unique (common are excluded) predicted enzymes (KOs) identified as DifRep for each KEGG pathway (top). Pathways that we refer to as “housekeeping” are grayed out. The asterisks highlight the metabolic pathways presented under each bar plot (see Table S9 & Suppl. Res. 2). At the bottom, **C** an overview of the predicted SCFA end products of the microbial metabolism. The MAGs that carry all the required KOs (manually verified) to produce the focal SCFAs are grouped by class. Arrowheads represent the number of predicted KOs required to produce each SCFA and are color coded to show the corresponding group of samples (summer or winter samples in our study; samples from calves before and after introduction of solid feed; [31]). Chemical compound illustrations (shown in hexagons) were obtained from PubChem (https://pubchem.ncbi.nlm.nih.gov/)

Looking at predicted enzymes, it appeared that MAGs carrying KOs related to the import of iron, phospholipids, methyl galactose, and others were more frequent in summer samples. In winter samples, KOs related to the import of aldouronate (which is released upon degradation of methylglucuronoxylan; a major hemicellulose present in plant cell walls; [40], glutamate and L-cysteine (Table S8; Sup. Res. 2B; ABC transporters). In general, predicted enzymes related to ABC transporters were the most common in both summer and winter samples (Fig. 4; Sup. Res. 2B), albeit importing very different compounds (Sup. Res 2B).

We then mapped the uniquely annotated KOs of each group in the 54 metabolic pathways that were identified as most relevant in the common contigs (80% of the samples; top 1000 in abundance). Restricting our observations to metabolic pathways for which all enzymes were present (in at least some of the MAGs) showed that the microbiome of “summer” samples presented more options for the import and metabolism of mono- and oligosaccharides, and these sugars likely originate from plant (mannose, cellobiose), fungal (chitin), and bacterial (MurNAc) cell walls (Sup. Res. 2B).

The uptake mainly of monosaccharides for fermentation — leading to the production of SCFAs — seemed to have a prominent role, as we found several predicted enzymes that could be assigned to relevant pathways (Fig. 4; Table S9). Commonly identified predicted enzymes with such roles were related to acetate metabolism and the production of acetoin and propionate through the 1,2-propanediol pathway [41]. We also identified several butyrate-producing taxa carrying almost the entire pathway for butyrate production except for a glutaryl-CoA dehydrogenase (Fig. 4; Table S9). However, this may be related to a misannotation as when we examined the KEGG pathway of *Butyrivibrio proteoclasticus* B316, a demonstrated butyrate producer, it had the same predicted enzymes as our taxa and it also appeared to be lacking the same dehydrogenase. Even though different MAGs can likely produce most of these end products in both winter and summer groups (Fig. 4; Table S9), the summer samples included a higher diversity of taxa carrying predicted enzymes that can facilitate such processes (Table S9). The remaining contrasts of functional pathways between summer and winter samples, with relatively high numbers of predicted enzymes, were related to stress response (quorum sensing, chemotaxis), bacterial secretion, oxidative phosphorylation, and the biosynthesis of cell wall components (Table S8).

We applied the same method to the twenty-four metagenomic fecal samples of young veal calves from a previous study [31], collected twice from the same cohorts (2 weeks old when fed only milk or milk replacer, and 2 months later, after switching to a mixed diet of milk and solid feed). Even though the double collections were not necessarily from the same animals but the same cohorts, these comparisons suggested that the introduction of solid feed changed the bacterial communities drastically (PERMANOVA *p* = 0.001), and the shift in diet explained approximately 23% of the variation while at the same time being raised in different farms had no significant effect (*p* = 0.055). Both alpha diversity and richness increased with the introduction of solid feed (Kruskal-Wallis Sobs = 13.23, *p* < 0.001; Kruskal-Wallis Shannon = 12.7, *p* < 0.001). A differential representation analysis concurred with the above contrasts, as more than 85% of the contigs identified as DifRep were from animals that were fed a mixed milk/solid feed diet, illustrating the higher diversity of the microbiomes of animals that were introduced to a solid feed. When looking at the DifRep contigs that were specific to the mixed milk/solid diet group, 81% of them came from 50 MAGs (median of predicted ORFs specific to the milk/solid group: 77%). Contrarily, only six MAGs had > 25% of their ORFs as DifRep, and only eight had > 10% of their predicted ORFs present exclusively in milk samples (Table S8).

We then examined the gene content of DifRep contigs and mapped the unique KOs of each group in the 54 metabolic pathways earlier identified in the common contigs of the intestinal microbiota (Table S6). This showed that in milk-fed animals, the KOs related to ABC transporters (uniquely present in that group) were related to the import of GlcNAc, glycine proline, and a few others (Sup. Res. 2C; ABC transporters). However, when the animals were fed a mixed milk/solid diet, we found uniquely predicted enzymes related to the import of more than 20 substrates (Sup. Res. 2C; ABC transporters). Besides the presence of features related to housekeeping pathways (e.g., ribosomal-related genes, suggesting an increase in diversity; see also “Discussion”), the introduction of solid feed increased taxa carrying ORFs related to the uptake and fermentation of sugars (Fig. 4; Table S8) and the potential production of SCFAs and AAs (Fig. 4; Table S9). Focusing once more on enzymes that were part of a complete pathway in some of the MAGs showed a bigger repertoire of enzymes related to mono- and oligosaccharide metabolism (Fig. 4; Sup. Res. 2) and potential SCFAs related production in the microbiome of feces from calves fed the mixed diet compared to calves fed only milk (Table S9).

### Virulence genes in the fecal microbiome and pathoMAGs

We set out to examine the virulence potential of the bacterial community present in the animal feces. Since the focus of our study was the cattle intestine, we focused solely on the six bacterial genera in animal feces that have been identified as major potential zoonotic and animal (henceforth referred to as pathogenic) threats. These include *Campylobacter*, *Clostridium*, *Escherichia*, *Shigella*, *Listeria*, and *Salmonella* and their known virulence genes, which included toxins, auto- and other transporter-related proteins, adhesion/attachment-related proteins, and flagella components [42–46].

**Fig. 5:**
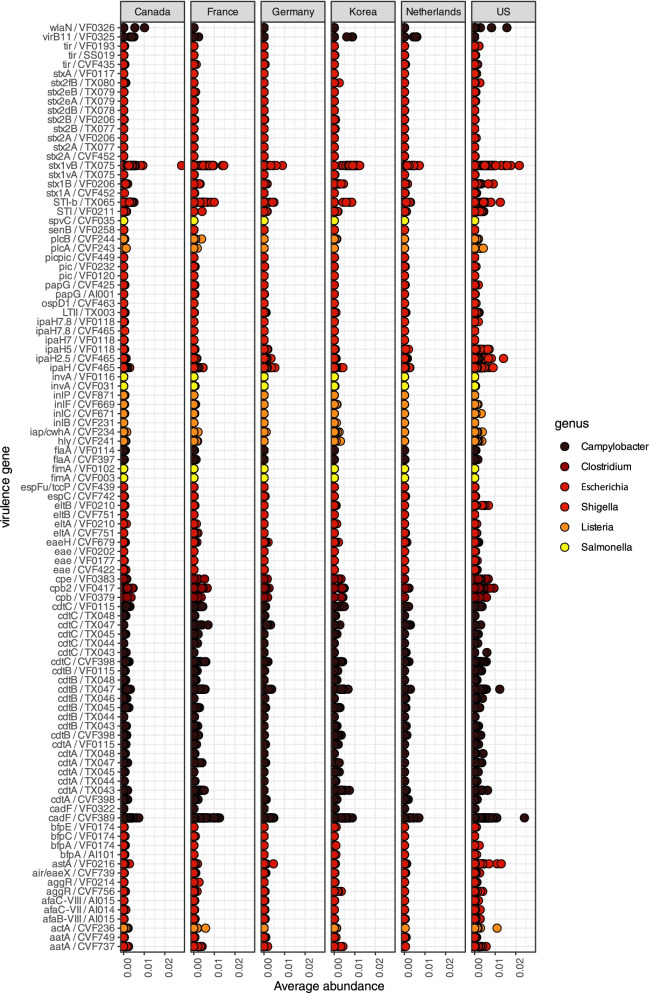
Occurrence of virulence genes in all 436 samples separated by country of origin. Color coding shows the six genera that we examined in our study (customized zoonotic VFdb; see “Methods”). Virulence genes are displayed to the left. Values are the corrected proportions of the identified VFdb features (counts of each feature/total counts of all features)

**Fig. 6:**
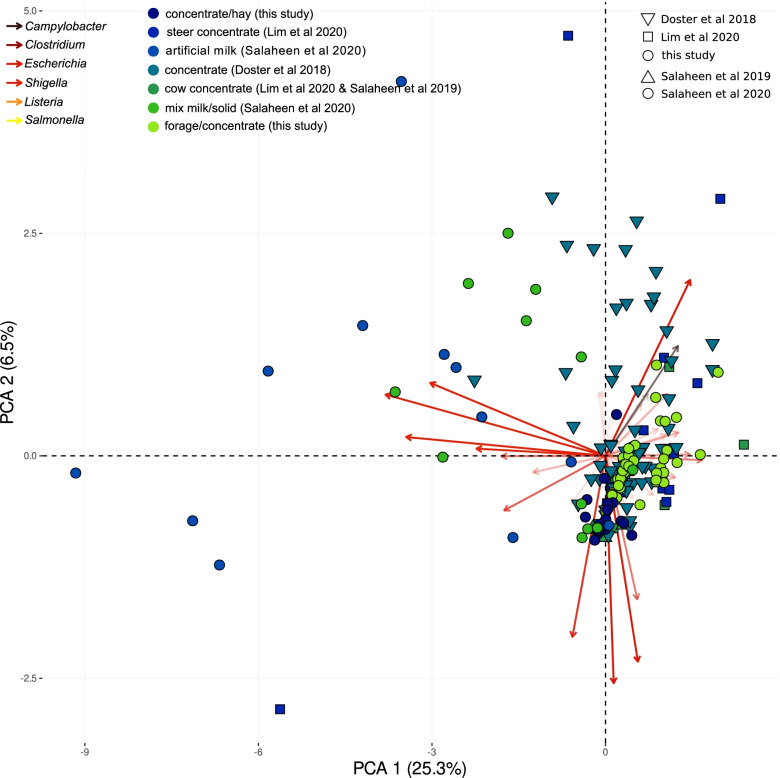
Overview of virulence genes in fecal microbiomes. Beta diversity of VFdb features in 208 feces samples of individual cattle. The ordination is based on a principal coordinate analysis (PCoA) of Euclidean distances among the CLR-transformed values of the corrected VFdb feature counts. Similar to Fig. 3, samples plotted are distinguished by diet (color) and study (shape), and the samples used for formal statistical comparisons ([31]; this study) are presented with filled circles. VFdb features, which are responsible the differences among samples, are color coded based on bacterial genus. Vector transparency is negatively associated with the contribution of vectors

We first performed an independent analysis to verify that identified virulence features of metagenomes can also be found in microbial isolates cultivated in vitro (Sup. Res. 3). We then took two approaches to examining the virulence potential of the fecal bacterial communities. First, we mapped the 150-bp length (non-assembled) filtered reads against our customized VFdb database, which contained the six focal genera. This showed that a total of 154 gene sequences were present to various degrees in animal samples across the six countries (of all 436 samples) but with the vast majority of them in low abundance (Fig. 5) and with features *cadF* (CVF389) and *stx1c *(TX075) followed by several *stx*, *ipaH*, and CDT (cytolethal distending toxin) encoding variants standing out as most prevalent. Considering that data came from eleven different studies, we did not compare the diversity across studies but instead restricted our formal statistical comparisons to samples from the same study, dairy cows during summer and winter in our study, and calves fed only milk and after being introduced to solid feed ([31]; Fig. 6). This showed that diversity was higher in samples coming from dairy cows collected in the summer (Kruskal-Wallis Sobs = 8.36, *p* = 0.004; Kruskal-Wallis Shannon = 9.3, *p* = 0.002), when comparing summer and winter samples, but no significant contrast was identified when young calves shifted from a milk to a mixed diet (Kruskal-Wallis Sobs *p* = 0.84; Kruskal-Wallis Shannon, *p* = 0.54).

We then examined the full-length predicted ORFs (contrary to the unassembled reads examined in the previous analysis) of the assembled contigs, which allowed us to identify entire virulence genes (and not just fragments of 150 bp) in the MAGs. When looking for these virulence genes at the six focal genera, 234 of them in 118 MAGs were identified with *Salmonella invA*, *Campylobacter wlaN* and *flA* being the most commonly found (Table S10A). However, when using all bacterial taxa (which allowed us to identify a much bigger diversity of those genes), hemolysin and hemolysin (*hly*)-like genes were clearly the most frequently present (Table S10B).

## Discussion

### What MAGs can and cannot say about their origin

We examined the microbiome of cattle feces, which mirrors that of the large intestine [47]. Compared to tissue sections, feces represent the microbial communities residing in the large intestine with sufficient accuracy [48, 49]. However, feces are more representative of the gut lumen and less of the gut mucosa [48]. This could explain why no mucinolytic enzymes were present in our metabolic reconstructions (Table S8; Sup. Res. 2), but GlcNAc import and metabolism-related enzymes, which is one of the main mucin components, were present. Also, considering that samples were collected from multiple or individual animals, and the pen floor, we would expect that not all MAGs are representatives of intestinal bacterial communities but also of bacteria originating from the pen floor and the proximate environment of the farm, and hence, for most of our analyses, we used the 210 samples coming from feces of individual animals.

In our study, we focused on the bacterial community, even though archaea, viruses, and Eukaryota sequences were present but in low abundance. This is not surprising since studies targeting prokaryotic 16S rDNA in cow feces have reported that following bacterial taxa, archaea are the second most prominent group but in low numbers [50–52]. However, this result is likely also affected by the underrepresentation of archaeal or other domain sequences in public databases like the NR, which we used in our study. We confirmed this hypothesis by mapping all sample reads to the archaeal contigs we assembled as well as the archaeal metagenome assemblies from a previous study [53]. This showed that using the latter as reference increased the number of identified archaeal reads, confirming our hypothesis (Table S3).

Our analysis used multiple samples from different studies (eleven studies including ours), and thus, confounding factors related to the methods of each study (i.e., DNA extraction, sequencing, analysis), the animals (i.e., gender, race, age), and the location have likely influenced the phylogenetic clustering of the MAGs. For example, sample type (pen floor or individual feces) could often explain MAG clustering (Table S5; Sup. Res. 1), and therefore, it is not surprising that the study ID often explained the MAG clustering of several bacterial classes (Sup. Res. 1; Table S5). This highlights the bias introduced because MAGs were built from samples of different locations and using different methods. The former can be eliminated by using a dereplicated MAG/genome set from multiple locations to build a global gene catalog that is more representative of the microbial community. However, for the latter, we would need to develop common protocols for community use, which would eliminate the “publication bias,” a crucial point that needs to be addressed [54].

The number of reference genomes used for a given comparison can impact the patterns of MAG clustering in phylogenies. If there are no closely related reference genomes, then the separation will appear more significant, a limitation that is impossible to overcome until more relevant genomes and MAGs start appearing in public databases. Our phylogenies attributed some MAGs as distinct from Hungate 1000 and RefSeq genomes (Table S5; Sup. Res. 1). Undersampling likely affects these patterns, making MAGs appear very divergent when compared to the genomes of close relatives, especially if there are not enough representatives (e.g., Fibrobacteria). However, that is less likely for classes like Clostridia in which MAGs formed distinct groups across all branches that contained fecal and non-fecal members and Bacteroidia (except for the *Prevotella* branches), the phylogenies with the most MAGs and genomes (Sup. Res. 1; Table S4). The distinct separation between rumen genomes and MAGs may also be the result of the physiological barriers among gut compartments as a result of the ruminant’s physiology [55]. A distinct separation among bacterial communities of GIT compartments has been demonstrated in previous studies [13]. The effect of undersampling also undermines our reference/core-MAG-based approach, used to quantify microbial diversity and abundance, an approach originally proposed by Wilkinson et al. [10]. However, our core set was already estimated to cover an average of 69.5% of the bacterial reads with a potential for this approach to slightly improve as more MAGs and genomes (from isolates) are added to this representative set.

### Functional insight into the biology of the large intestine

When looking at the “common” sequences (Table S6), besides housekeeping genes (i.e., ribosomal, membrane synthesis, and auxotroph lifestyle related), the most commonly found ORFs were linked to transporters. Transport is a key process that facilitates survival and competition as it allows scavenging compounds/micronutrients that cannot be synthesized, secretion of deleterious compounds, and other [56]. Many of the common predicted enzymes (Table S6; Sup. Res. 2A) were linked to such roles as we identified a large repertoire of predicted enzymes facilitating scavenging, like the Pst transporters, which aid in the assimilation of phosphate [56], and the AfuABC suggested to be critical to sugar-phosphate uptake [57]. Secretion system-related enzymes were often linked to the Sec and Tat protein export systems [58], the latter known to facilitate iron scavenging [59], pathogenicity [60], phosphate acquisition [61], and motility and resistance to AMPs, heavy metal, and H_2_0_2_ [62]. Flagella-related genes, typically associated with motility that allows bacteria to reach target niches and contribute to pathogenesis (as these roles have been mainly studied in pathogens [63]), were present in the highest copy numbers (compared to other VFdb features) and were identified as an influential feature separating classes (Fig. 2; Table S7B). Finally, the ectoine biosynthesis pathway, found intact in our MAGs, may be key to the survival of bacterial taxa in the cattle intestine environment. Ectoine is an osmolyte that helps bacteria to cope with fluctuations in osmolarity [64]. The large intestine together with the omasum are the main parts of the GIT where water absorption takes place [12]. This suggests that osmolarity fluctuations in these niches are commonplace, which would justify the presence of genes linked to ectoine production or glycine betaine uptake (*opuA* may have putatively emerged in Clostridia, Fibrobacteria, Negativicutes, and Spirochaetia taxa; Table S7A; [65]).

The other functional categories that were found among the most commonly encountered predicted genes (Sup. Res. 2) were linked to carbohydrate and AA metabolism, both in the means by which these compounds are transported across membranes as well as their metabolism. Metabolism-related predicted enzymes were also the most influential features differentiating taxa (Fig. 2; Table S7B). When we looked exclusively at KEGG complete pathways with the most enzymes combined, most were linked to carbohydrate catabolism (Fig. 4; Sup. Res. 2). Carbohydrate metabolism had a prominent role both when looking at common sequences (Table S6) and the contrasts between dietary transitions (Table S8). In most cases, this involved the catabolism of mono- and oligosaccharides to produce energy and the likely production of SCFAs [14]. This is far from surprising as complex polysaccharide breakdown occurs mainly in the rumen [8–10], while the bacterial community of the large intestine contributes to the final step of the digestive process, degrading part of the undigested feeds and fermenting mono- and oligosaccharides [12]. SCFA production takes place across the entire cattle GIT, spiking in the rumen and the large intestine [14].

Anaerobic fermentation, however, does not only serve the energy requirements of the microbial symbionts. In animals and humans, large intestine microbial symbionts produce SCFAs that the host cells absorb [14, 66–68]. Besides the obvious energy gain, SCFAs absorption has diverse benefits for the host such as promoting the maintenance of intestinal barrier integrity, stimulating the production of mucus by the epithelial cells, and modulating the immune response [69, 70]. The presence of acetate-fermenting enzymes (Sup. Res. 2) suggested the existence of taxa that may be able to utilize this common end product of microbial fermentation [14, 71] and/or taxa with the ability to alternate between production and fermentation of acetate, a switch that is often dependent on the acetate concentration in the environment [72].

The complete pathways of lysine and threonine production were found in several MAGs (Table S9) and many enzymes in the common contigs (Sup. Res. 2A). Considering that lysine and threonine are among the top three limiting AAs in cattle feed, this discovery could suggest a potential microbial service to the host. When looking at the list of MAGs with the potential of producing lysine (Table S9), MAGs similar to *E. coli* and *Corynebacterium efficiens* taxa are of special importance, not only because they had a complete pathway (as this is not proof of exporting these common goods) but also because they have been identified as promising lysine producers in in vitro cultures [73]. In addition, the presence of a threonine aldolase in multiple fecal-associated taxa (Table S7A), which further facilitates the conversion of threonine to glycine, may be linked to channeling resources to the production of glycine (depending on the needs of the microbial symbionts or the host).

The other complete pathways that were found in fecal MAGs involved spermidine and putrescine metabolism (Table S6C). These are polyamines that may have an anti-inflammatory effect in the gut and help with numerous other physiological processes [74]. The putrescine and spermidine production may be linked to the complete arginine production pathway found in several MAGs since arginine is a precursor of polyamines, suggesting that the potential arginine-producing and polyamine-producing MAGs in the large intestine (Table S10) may be metabolically interdependent.

Finally, microbial cobalamin production by the rumen microbiota has been suggested as a potential symbiotic function [8], but none of the fecal MAGs had the potential to fully produce it (even though often the pathways were almost complete), which also applies to thiamine, a vitamin that has a critical role in dairy cattle health as its deficiency leads to disease [75]. However, considering that cobalamin has a crucial role both in rumen and the large intestine and that no taxa encode all necessary enzymes, the incomplete pathways may signify that its production is the result of the complementary contributions of multiple symbionts [8, 76].

### Dietary changes affect the microbiome

The decrease in microbiome diversity as a result of decreasing forage and increasing concentrate has been previously demonstrated in the cattle intestine [77, 78]. The increase in readily fermentable carbohydrates in mixed diets has been shown to reduce the pH in the rumen and the large intestine, which makes the environment less favorable for many microorganisms, reducing the overall richness and diversity of microbial communities throughout the digestive tract. The diversity of VFdb genes was reduced dramatically after the animals in our study moved indoors. Seasonality is common in infectious diseases, with a higher incidence of pathogenic *Salmonella* and *E. coli* O157 and non-O157 in cattle during warmer months [79, 80]. Similarly, the concentration of nonpathogenic *E. coli* in the rumen and feces of feedlot cattle is typically greater when the animals are on corn grain than on hay diets, while the duration of O157:H7 shedding is longer on forage diets [81].

The season has been shown to impact the microbiota composition of the GIT as revealed in feces [82]. Similarly, the decrease in O-antigen nucleotide sugar biosynthesis predicted enzymes is likely linked to a decrease in taxa producing O-antigen [83] and the MurNAc uptake linked to auxotrophs (e.g., [84]. These changes could probably be explained by the reduced diversity of the microbiota from summer to winter (Table S8). Energy and metabolism wise, the switch from summer to winter diet changed the SCFA and AA production potential, since even though the enzymes were present in both groups, they came from different microbes (Table S9). Since previously it has been suggested that SCFA production is drastically affected by the forage to concentrate ratio in the rumen [85–87] and the feces [85], and considering that we also observed big differences in taxa-producing SCFAs linked to taxa substitutions, our results imply a similar effect. Therefore, it would be of great relevance for future studies to investigate which bacterial substitutions are linked to increased SCFA production and whether these changes are due to increased abundance or replacement with more efficient producers.

When we looked at the Salaheen et al. [31] twenty-four samples, the introduction of solid feed induced a massive increase in diversity, also shown by 16S [31] and similarly observed in previous studies [88]. The observed increase in O-antigen- and lipopolysaccharide-related genes likely represented an increase in diversity (Fig. 4), since some microbial taxa with reduced genomes have lost various ribosomal genes that do not impede their growth [89]. The change in diversity was also roughly visible when we examined the contig assembly characteristics, which showed that preweaned calves returned proportionally more bacterial contigs than the rest of the samples, suggesting the absence of other than bacterial domains (Table S3A). Looking at the metabolism, the introduction of solid feed resulted in a decrease in predicted enzymes linked to transporters of acetylglucosamine and proline, substrates abundant in milk and milk replacers [90], a large increase in sugar transporters and catabolism-related enzymes (Sup. Res. 2C), and an increase in SCFA- and AA-related enzymes (Table S9).

### Host selection

Microbes associated with animal hosts, like gut symbionts, are under constant host selection [91], as hosts will favor symbionts providing valuable services and select against transient opportunists and pathogens. Yet this rule is applied somewhat differently in invertebrate (e.g., insects) and mammalian host systems, primarily because of the massive diversity of the latter [92]. The high complexity, resulting in increased competition, does not allow for strong interdependence between symbiont strains and host and results in a more relaxed host selection and the frequent acquisition of environmental microbes [92], as opposed to invertebrate systems in which nutritional mutualisms are strongly correlated with vertical transmission [93]. The result is an observed functional redundancy as hosts can reap symbiotic benefits that are not tied to certain symbionts but substitutes (other microbes) that happen to be present at the time. The benefits are often linked to the production of common public goods (end- or by-products of microbial metabolism), thus resulting in frequent symbiont substitutions [94].

The lysine/threonine and arginine production (as a result of aspartate catabolism) could be an important trait of the microbiota — simply because it is universally present in all samples — even though its production is allocated to different symbionts across samples. This is also the case for SCFA production as comparisons showed that such pathways survived the dietary changes via shifting production across different MAGs (functionally replacing the previous ones; Table S9). Even though it is less surprising, as the production of SCFAs by gut microbes is a universal trait, possibly present in all animals,  including cattle  [85].

## Conclusions

Our observations add significant insight into the complexity of the relationship between cattle and their large intestine microbes. In addition, our study generated a global MAG catalog that may cover up to almost 70% of the intestinal bacterial symbionts and can be used for future studies. However, MAGs are artificial proxies for actual large intestine microbiomes obtained from fecal samples and therefore offer only a representation. Some of the potential replacements observed (Table S9) may be the result of low sequencing depth as our metagenomic approach has likely not unraveled the entire microbial diversity of the feces. Follow-up work via targeted functional approaches and in vitro assays are needed to further examine these intriguing relationships in depth.

## Methods

### Animal rearing, collection of fecal samples, DNA extraction, and sequencing

We collected fecal samples from lactating Holstein dairy cows raised in the experimental farm of the INRAE Herbipole Unit (UE 1414) in Theix (Saint-Genes-Champanelle, France), in September and December 2020. In September (summer regimen), the animals spent the day grazing in a permanent grassland near the farm, complemented by 3 to 5 kg per day of a ration made of corn and grass silage and hay; in December (winter regimen), they spent the entire day indoors, with ad libitum access to a standard diet made of 30% corn silage, 25% grass silage, 15% hay, and 30% concentrate (see Table S1 for details). We sampled 48 cows, collecting fecal samples from 26 of these only in September and from 22 both in September and in December (leading to 70 total fecal samples). We collected feces by filling a 50 ml falcon tube (ca. 15–40 ml of material) either by sampling from the top layer of freshly (within 2–3 min) deposited feces using a sterile plastic spoon or by directly putting the falcon tube under defecating animals. Upon collection, we immediately put samples on ice, transferred them to the lab, and placed them at −80 °C, after aliquoting them in 1.5 ml Eppendorf tubes. We extracted DNA from approximately 250 mg of each sample using the Quick-DNA Fecal/Soil Microbe Miniprep Kit (D6010 — ZYmoResearch), evaluating the quality of extracted DNA via a Nanodrop spectrophotometer before sending them to Novogene (Singapore) for sequencing. Library construction was performed using the NEBNext Ultra II DNA Library Prep Kit, and all samples were sequenced in two separate runs in a NovaSeq 6000 to obtain a minimum of 60 million reads per sample.

### Filtering of reads, assembly, binning, and taxonomic characterization of contigs

In addition to the 70 samples that we sequenced ad hoc for this study, we also downloaded 366 additional metagenomes originating from individual cows, bulls, or calves [25, 28, 31, 32] and composite fecal or pen floor samples [26, 27, 29, 30, 33, 34], summing up to a total of 436 samples. For each sample, we trimmed and filtered raw reads first using Trimmomatic [95] to remove adapters and then bowtie [96] and the latest Bos taurus RefSeq assembly (GCF_002263795.1) to remove cow reads. We assembled filtered reads using megahit (v1.1.1) [97], via the option “−meta-large” and a minimum 300 bp contig length. For the binning process, we used the Autometa pipeline [98] to first map the filtered reads to the assembled contigs and create a coverage table and then to identify the ORFs via prodigal [99], compare them to the NCBI nonredundant (NR) protein database (downloaded December 2020) and assign taxonomy to each contig.

### MAGs

To create the MAGs for our study, we used the Autometa pipeline with small modifications (for an overview of the pipeline, see [98]). In short, following trial runs using cutoffs of 300 bp, 500 bp, 750 bp, and 1000 bp as contig length cutoff, we ran the “run.autometa” command for all 436 samples individually using only contigs of 1000 bp or longer (using contigs of 750 bp did not improve or increase the number of MAGs produced, while runs using 300 and 500 bp cutoffs failed because of very high computational demands). We evaluated the contigs from the identified MAGs using checkM (v1.1.3) [38]. This showed that out of the > 30,000 MAGs, 2114 MAGs had > 85% completeness and < 5% contamination.

Using the predicted ORFs from the 2114 MAGs, we identified single-copy orthologs via orthofinder [100]. To determine close relatives of the assembled MAGs, we compared their predicted ORFs’ AA sequences to those of 400 bacterial isolates from the rumen (Hungate 1000 project; [8]) and the predicted ORFs’ AA sequences of 20,584 complete bacterial genomes available in NCBI (RefSeq March 2021). In short, for our comparisons, we split the MAGs based on the bacterial class that they belong to (based on the Autometa classification), and we chose as query a representative set of MAGs: the 149 MAGs that were identified in the samples sequenced in this study. For the Bacilli, Coriobacteriia, Elusimicrobia, and δ- and ε-Proteobacteria classes, which had no representative in the 149 MAG dataset, we used all MAGs belonging to these classes from the 2114 MAGs dataset as a query. We then created diamond BLAST databases [101] using the Hungate 1000 and the RefSeq collections, which we used to compare the AA sequences of the predicted ORFs from the MAGs produced in our study (*E*-value = 1e-50, min percentage identity = 70%). This identified 34 Hungate 1000 isolates’ genomes whose ORFs were in the top hits (best match). We followed the same BLAST strategy using the RefSeq collection. Using the 2114 MAGs sequences and the checkm information about their completeness and contamination, we created a dereplicated set of the MAGs with default options (primary clusters cutoff: 0.9, secondary clusters cutoff: 0.99) via dRep (v3.0.1) [102]. To evaluate the percentage of diversity captured by the MAGs across samples, we created a reference database and aligned the trimmed and quality-filtered reads of each sample using bowtie2 [103] and the 1232 MAGs nucleotide sequences. We also performed similar comparisons using as reference the assembled contigs of each sample.

### Phylogeny

For the phylogeny reconstruction, we used 15 (out of the 19) bacterial classes that we identified above (based on the diamond BLAST comparisons), excluding the Elusimicrobia, ε-Proteobacteria, Chlamydiia, and Lentisphaeria for which we had only six, four, one, and one MAGs, respectively. Using the diamond BLAST results, we classified each MAG based on the top hits that their predicted ORFs gave (i.e., utilizing the most commonly present taxon in the BLAST results for each MAG). Out of the 15 bacterial classes, Bacteroidia and Clostridia were the ones with the most members (568 and 398 MAGs, respectively). For each class, we identified single-copy orthologs for the respective MAGs as well as their close relatives from the Hungate 1000 isolates and RefSeq complete bacterial genomes, via orthofinder. We excluded RefSeq genomes with peptides marked as “MULTISPECIES.” To construct phylogenies, we then focused on the single-copy orthologs conserved across MAGs and complete genomes. Although straightforward for most classes, this process required manual curation for Clostridia, Bacteroidia, and Actinobacteria. For these classes, we employed the following strategy:

i) Prioritized MAGs that had > 90 completeness and < 3% contamination and eliminated the ones that did not fulfill these criteria if their removal increased the number of single common orthologs.
ii) We used the number of multiple copy and absent orthogroups (predicted orthologs) of RefSeq and Hungate 1000 genomes as reference for further excluding low-quality MAGs. We evaluated the MAGs based on the sum of multiple copy ORFs and absent orthogroups and excluded MAGs that more than 10% of their orthogroups had multiple copies or were absent.

We extracted the AA sequences for each of the fifteen classes, which allowed the construction of gene specific alignments using MUSCLE v3.8.31 [104]. We further refined these alignments using the trimAl software, which removed all positions with gaps in 10% or more of the sequences unless this left less than 50% of the original sequences [105]. The filtered alignments were further tested for recombination using the PhiPack software [106]. For the remaining genes, we concatenated individual AA alignments using Amas 0.98 [107] and selected the appropriate substitution models after testing them with ProtTest v3.4 [108].

We used the refined AA alignments for each class to reconstruct maximum likelihood (ML) phylogenomic trees. We used RaxML v.7.3.0 [109] with specified partitions of the concatenated alignments, which produced 25–128 partitions (one partition for each gene) for each alignment. For δ-Proteobacteria, for which we identified 165 nonrecombinant orthogroups, we further removed the 37 orthogroups (ORFs) of the smallest length, as RaxML is optimized to run a maximum of 128 gene partition models. For the *Escherichia* phylogenetic tree exclusively (for which we wanted to increase the phylogenetic resolution as much as possible), we changed the RaxML parameters to run a phylogeny of 843 genes. We used each alignment and the accompanying partitions information to run: (i) 1000 bootstraps using the LG + IG rate heterogeneity model [110] and the rapid bootstrapping algorithm and (ii) an ML analysis using 20 replicates to construct a “bestTree.” When both analyses were finished, we used the bootstrap support values to draw bipartitions on the best ML tree (support values assigned to branches of the tree). Each tree was then imported into R and was further annotated to produce figures using the ggtree package [111] to create the additional panels showing the country of origin (based on the sequencing data the MAG came from) and the average abundance. The latter was calculated by first obtaining the sums of the corrected values of the abundance of their contigs (calculated by KMA) and then estimating the means across all samples.

Using the protein alignments produced, we transformed the protein alignments to matrices using the “phangorn” package in R and performed PERMANOVA tests using 1000 permutations to examine the correlation between MAG dissimilarities and their origin (the study the samples came from) or sample type (the source material the samples came from; individual, composite, pen floor), by performing two independent tests for each alignment. All *p*-values were fdr corrected.

### Functional annotation

Functional annotation was performed using the eggNOG-mapper (version 2.0.4) [39]. In short, we used the AA sequences of the predicted ORFs identified by prodigal (integrated into the Autometa pipeline, see above) as input to perform a diamond BLAST (v2.0.5.143) against the eggNOG protein database (v2020-11-12), with a 1e-05 E-value as cutoff. We used the best match of each query as input for the eggNOG-mapper and subsequently imported output tables to RStudio for further processing and multivariate analyses. The KOs were used as input to render the predicted enzymes on KEGG maps using the “pathview” package in R. We took two approaches to identify complete pathways in our MAGs: (1) we examined which of the 450 KEGG modules (https://www.genome.jp/kegg-bin/get_htext#B1) were complete in the 1232 core MAGs, and (2) we examined manually (using the “ pathview” package in R and visually comparing), all metabolism-related pathways that had more than 10 enzymes present in the common contigs (Table S6) and all metabolism-related pathways presented in Table S8.

### Gene content analysis

To identify distinct gene gains and expansions across MAGs, we first set a midpoint root in the phylogenetic trees generated with RaxML using the figtree software (v1.4.4). Then, trees and feature tables that contained the KEGG functional annotation information obtained by the eggNOG-mapper were imported in the Count software [112]. Using Count, we performed a gene content analysis (Wagner parsimony analysis) to identify the potential emergence of novel gene families. The method infers family- and lineage-specific changes along the branches of a phylogenetic tree by performing ancestral reconstruction. We applied this method to the 15 class-specific phylogenies we constructed with RaxML and the KEGG annotation. However, considering that we used MAGs, we focused only on gene gains and expansions on fecal MAGs (i.e., KOs that were uniquely higher along the branches of fecal MAGs).

### Sample correction

Since microbiome data are compositional [113], for all downstream comparisons across samples, we followed the following strategy:

i) Using the most recent SILVA database at the time of the analysis (v138.1) [114], we constructed a kma 16S reference database of only bacterial sequences and used it to map the reads of each sample (*n* = 436). The fragment count numbers generated were used for correcting each sample and transforming the counts to proportions (contig-of-interest/gene-length)/(sum (16S SILVA gene counts/16S gene length)).
ii) For the pathobiome carriage, we used the VFdb database (see above) and kma to align the filtered reads to the database. We then took the ratio of the fragment counts of identified pathogenic genes of interest (e.g., *stx*, *eae*) to the total counts of VFdb features (generating proportions of pathogenic genes/total pathobiome).

Datasets were then imported in RStudio (v1.4.17.17) operating R (v4.1.0) where 0 s was imputed using the “zCompositions” package [115]. The resulting tables were CLR transformed using the “clr” function in the package “compositions” [116], and data calculations were performed using Euclidean distances. The only exception was the differential representation analysis in which we used the corrected, but not CLR transformed data.

### MAG and VFdb abundance tables construction

Using the reduced dataset of 1232 MAGs, we created a reference template with kma v1.3.11 [117], which we used to map reads from all samples. To do so, we first used “kma_index” to build a reference template using the 1232 dereplicated MAGs and then mapped each sample using the options “-mem_mode,” “-ef,” “-1t1,” “-cge,” and “-nf.” Similar to the MAG abundance table construction, we created a reference template using the latest version of the full nucleotide sequence dataset within the VFdb database (downloaded on the 11th of February 2021). The fasta file was used as input for the kma_index command, and using the same options as above, we aligned all filtered fastq files (cow sequences removed) to the VFdb reference template. For the VFdb database, we created a subset focusing on the most common zoonotic and animal genera and their virulence genes (see below).

### Principal coordinate ordinations, matching PERMANOVAs, and other stats

Corrected, imputed, and clr-transformed data tables (VFdb and MAG) were used as input for principal coordinate analyses using their Euclidean distances and the package vegan in R (v4.1.0, [118]. For ordination, we only used samples coming from individual animals and for which we has information about the animal’s diet. This reduced the number of samples to 208. We also calculated the variance of the first two axes and identified the most influential species (VFdb or MAGs features) in R and plotted ordinations using the packages ggplot2 [119] and factoextra [120]. To examine significant differences between groups, we used PERMANOVA (permutational multivariate analysis of variance) based on the pairwise dissimilarity matrices with the “adonis” command in vegan, using 999 permutations.

To identify MAG contigs that were differentially represented across samples, we used the nonparametric “indicspecies” package in R [121]. Samples sequenced in this study and samples from Salaheen [31] were used as input (corrected and rounded but not imputed and not clr transformed) in two independent analyses using the multipatt function and 1000 permutations. Samples were separated either as summer and winter (this study) and before and after the introduction of solid feed [31]. The obtained pvalues were fdr corrected via the package “stats” in R. For each significant differentially identified (*p* < 0.05) contig that was assigned to one of the treatment groups (stat > 0.5), we extracted and analyzed the unique in each group predicted KOs.

### Pathobiome definition and virulence gene analysis

For virulence characterization, we focused on foodborne pathogens that have caused (or have the potential to cause) epidemics [122]. Therefore, we focused on the genera *Campylobacter*, *Clostridium*, *Escherichia*, *Listeria*, *Salmonella*, and *Shigella*. The VFdb database contains also nonvirulence-related genes (originating from pathogens, but not necessarily linked to pathogenic traits) often highly conserved and therefore very similar to orthologs of nonpathogenic strains. Therefore, we focused only on genes with documented virulence, based on the most up-to-date knowledge as follows:

- For *Campylobacter jejuni*, *flaA*, *cadF*, *iam*, *cdtABC*, *virB11*, and *wlaN* [46]
- For the *Escherichia* genus, ETEC: enterotoxins, heat stable toxins STa and STb, and heat labile toxin LT; STEC: *stx2*, *stx1*, and *eae*; EPEC: Intimin (Eae); DAEC: *afaBC* (Afa/Dr); EAEC: *aaiC* and *aatA* [aggregative adhesion-encoding plasmid in EAEC was detected using the classical CVD432 probe (AA probe)]; and EIEC: *ipaH* [44].
- For *Clostridium perfringens*, *cpA*, *cpB*, and *cpE* [43]
- For *Listeria* monocytogenes, *inlA*, *inlB*, *hly*, *plcB*, *plcA*, *actA*, and *iap*
- For *Salmonella*, *invA*, *fimA*, *stn*, *spvC*, and *spvR* [42]
- For *Shigella* ShET (same as enterotoxin), *stx*, *ipaH* enterotoxin, and pic [44, 45]

We used the MAGs bacterial ORFs as input to compare to the customized VFdb database including only the six pathogenic genera and their virulence genes. We performed comparisons using diamond BLAST on the AA sequences, with an *E*-value of 1e-15, a minimum query coverage of 70%, and a minimum identity of 35%. We extracted and aligned ORFs that gave significant matches using MUSCLE v.3.8.1551 [104]. We trimmed alignments using trimAL [105] and built a distance matrix for each alignment via the “distmat” function of the EMBOSS package [123]. We imported distance matrices in R where we calculated and plotted means, medians, and number of observations/occurrences via ggplot2 [119]. We performed nonparametric statistical tests between medians or occurrence and sample type (composite vs. individual), location, animal type, and farm size using the R “kruskal.wallis” test. We used only the two subsets for which we had two groups of samples fed on different diets coming from the same study (i.e., the current study and [31]). *P*-values were fdr corrected.

## Supplementary Information

**Additional file 1: Table S1.** Shotgun metagenomic data used in this study. From left to right: accession and bioproject numbers, study, location, sample type, diet, age, farm id and size (whenever available). The table is also accessible at www.fecobiome.com/resources/data by entering the keyword “shotgun” in the “search our database” box. Samples can be further filtered by location or farm name (e.g. using the keywords «Theix» or «Saint-Gene-Champagnelle» will only list the samples sequenced in our study).

**Additional file 2: Table S2.** Summary of the contigs assembly. From left to right: sample name (accession number), number of contigs and number of base pairs, minimum, maximum and average contig length, N50 and mean coverage of contigs (estimated by mapping filtered reads to contigs using Bowtie2).

**Additional file 3: Table S3.** A: Summary of the assembled contig classification: Number and proportion of contigs assigned to each domain, for each sample. The absolute figures are shown at the left and the percentages at the right. The average percentage for each domain is also presented in the small subtable to the right. B: Archaeal sequences abundance estimate: Number of reads properly paired to the 155 RUGs previously presented [53] and the assembled contigs identified as archaea in our study. The absolute numbers and percentages are presented for each sample. Mean percentages and medians across all samples are presented to the right.

**Additional file 4: Table S4.** Overview and characteristics of the 2114 MAGs, the 74 Hungate and the 102 RefSeq genomes used to reconstruct phylogenies in our study. From left to right: the internal code (a derivative of the accession number the data came from and the autometa id); the alternative name, which we either generated by identifying the closest match following BLAST comparisons with RefSeq or the assigned strain name (for reference genomes downloaded from databases); the accession number the MAG came from (when relevant); country where the sample was collected; author; dRep clustering and checkm (estimated completeness and contamination) and mean contig coverage (estimated by mapping filtered reads to contigs using Bowtie2). The overall mean and median are presented as well. The MAGs with the same drep clustering id are >99% identical. The last column indicates the 1232 MAGs that were selected for the dereplicated dataset. The sequences of the 2114 MAGs are available here: doi.org/10.15454/UIJTJA. B: Mapping of filtered reads to contigs and the core MAGs. From left to right: Sample names, the total number of reads for each sample and the number of reads successfully mapped to contigs, the percentage of the number of reads mapped to contigs, the number of reads mapped to MAGs, and the percentage of reads mapped to MAGs. The smaller subtable to the right presents: the average percentage of reads successful mapping to contigs and to MAGs (top); the average percentage of reads mapped only to bacterial contigs (based on the classification from Autometa; NR database); the percentage of bacterial reads (based on the bacterial contig estimate) mapped to MAGs.

**Additional file 5: Table S5.** PERMANOVA tests examining the impact of the different study and sample type (in two independent studies) on the fifteen class-specific reconstructed MAG phylogenies. Dissimilarity matrices were created using AA alignments, built from the phylogenies presented in Sup. Res. 1. Two independent PERMANOVA tests were performed on each matrix, using the study identifier (author) and sample source type as explanatory variables.

**Additional file 6: Table S6.** Common KEGG metabolic pathways in the microbiome of cattle feces. A: List of KEGG pathways along with the 175 pathways (marked with an asterisk) we chose to examine as relevant (see Methods) for our analyses. B: Most common pathways based on contigs present in at least 80% of individually collected fecal samples (*N* = 210) and the most abundant 1000. From left to right: the metabolic pathway KEGG id, the number of predicted KOs identified in that pathway, the pathway name, additional notes inferring the function of their study based on our analysis, and the MAGs carrying most enzymes in the common contigs. C: KEGG modules complete in at least one MAG. For this analysis, the 450 predicted modules (KAAs) were examined against the dereplicated set of our core MAGs and we present the modules that were complete (all their enzymes present) in at least one MAG. From left to right: the module description (including the KEGG pathway/s it is linked to), and the KEGG subcategory and category it belongs to.

**Additional file 7: Table S7.** Count software analysis of the 15 taxonomic classes (A) and list of the most influential KOs (B) identified by the principal coordinate analysis presented in Fig. 2B. A: From left to right: the KO id, the number of gains and expansions for the given ortholog (overall) and their the sum, the gains and expansions in RefSeq genomes, the gains and expansions in fecal MAGs and the taxonomic class the analysis was conducted for. In the last two columns, the overall gene losses and contractions are presented as well but are grayed out on account of not examining them because we used MAGs. On the smaller subtable to the right, we present the KOs that were identified multiple times in the independent Count analyses (we performed 15 analyses one for each class that we built a phylogeny). B: From left to right: the KO id, the coordinates and the contribution of each feature based on which we selected the top twenty (as most influential) to present in Fig. 2. For the top hundred most influential vectors (a.k.a. features) we present the KO description as well. Bold font indicates features that were identified both in the Count analysis for the Bacteroidia, Clostridia or Spirochaetia classes and among the top 100 most influential features in the PCoA analysis.

**Additional file 8: Table S8.** Overview of the comparisons between groups of samples to identify differentially represented (DifRep) metabolic features. The four sheets present comparisons for the dairy cow samples coming from Theix (our study) separated in winter (Theix-winter) and summer samples (Theix-summer); and for the calf samples coming from the Salaheen et al. [31] study, separated into samples from animals fed exclusively milk (Salaheen-milk) and samples from calves that after the introduction of solid feed in their diets (Salaheen-solid). For each tab, from left to right, we present the metabolic pathway id; the pathway description; the number of enzymes of that pathway that were identified in the focal contigs. The rest of the columns present the number of predicted enzymes discovered in group-specific (winter-specific, summer-specific, solid-specific or milk-specific) MAGs identified by our analysis (i.e. MAGs that had >25% -for summer or solid group- or >10% -for milk or winter group- of their contigs DifRep; see also Results).

**Additional file 9: Table S9.** Distribution of metabolic functions with potential mutualistic benefits for the host in the groups of samples used for our formal comparisons and the common sequences. In each tab, we present the MAGs that contain relevant enzymes (based on KOs annotation) and the sum of the predicted enzymes identified under the column ‘total’. If the MAG (and by extension its enzymes) is also present in one of the focal groups of samples (i.e. [31]; milk or solid group, this study; summer or winter samples and the common sequences) this number also appears under the respective column (milk, solid, winter, summer or common). MAGs that we manually verified that carry all predicted KOs for the production of an endproduct (by visually inspecting to confirm the completeness) are highlighted in bold font. In each tab, we also present (to a small subtable to the left) the number of MAGs identified carrying enzymes in the focal groups of samples. In the acetate tab, we also present the MAGs that carry the predicted EC enzymes that would be required for acetate production (for an overview see Figure 1 of [124]).

**Additional file 10: Table S10.** Overview of virulence genes detected in the 2114 MAGs using the virulence genes of interest in the six pathogenic genera (A) and in all taxa (B). From left to right: the percentage, E-value and bitscore of diamond BLAST comparisons, the VFdb identifier, the VFdb preferred gene name, the VFdb taxon, MAG name and alternative name, the metagenomics sample, the country and the study the MAG came from, the dRep cluster identified and the average abundance of the MAG in the 436 samples. The subtable to the right presents the gene prevalence based on occurrence (the times a VFdb gene gave a positive match to the dataset).

**Additional file 11: Supplementary Results 1.** Phylogenies built in our study on the basis of 13-843 AA sequences of single copy orthologs. The first fifteen panels present the class specific phylogenies for the 2114 MAGs identified in our study and their close relatives. Bootstrap support is presented (light gray next to branches) and colored circles show study ID of the sample that each MAG was generated from. The light gray and dark gray circles show genomes from the reference databases (Hungate 1000 and RefSeq respectively). At the upper right corner of each phylogeny, the class and number of genes used for concatenated alignments are presented. Single asterisks next to names mark MAGs that originate from fecal samples that were collected from single animals and double asterisks MAGs that originate from fecal samples collected for this study. Average abundance for each MAG was calculated by first summing the abundance of all contigs of a MAG per sample (calculated by KMA) and then by estimating the mean across all samples. The last two panels present the *Escherichia* specific phylogeny built using MAGs built in our study and *Escherichia* isolates sequenced in a previous study [23] and a global phylogeny (labeled as «common tree») built using 87 high quality MAGs (>99% completeness), which are representatives of most bacterial classes. Signs with exclamation marks in the phylogeny of *Escherichia* mark isolates carrying virulence genes. Bacterial classes are displayed with a red font in the ‘common tree’ phylogeny.

**Additional file 12: Supplementary Results 2.** Summary of the most common KEGG metabolic pathways illustrations (which are complete in at least one MAG). Pathways are presented for the common (80% of 210 individual sample and most abundant; Table S6) contigs (A), the contrast between winter and summer samples in our study (B) and the contrast between animals before and after introduction of a solid feed (C) (Table S8). The illustrations present the parts of the metabolic pathways that show specialization (i.e. import of compounds) and not common/housekeeping parts (e.g. catabolism of glucose to pyruvate or the Entner–Doudoroff pathway) for simplification. In addition, parts of the pathways that are likely linked to housekeeping (e.g. cell wall biosynthesis), pathogenesis and other non-metabolic functions have been omitted.

**Additional file 13: Supplementary Results 3.** There are two main approaches to identify zoonotic and animal pathogens in fecal (or other) samples: a culture-based approach that typically isolates microbial strains and then examines their virulence potential using PCR or sequencing (individual genome sequencing) and a shotgun metagenomic approach that examines all sequencing reads against a set of known virulence genes (e.g. VFdb database). A common controversy between the two methods is that the metagenomic approach likely overestimates the diversity of community members and the diversity of virulence or other microbial genes, while the culture-based approach underestimates it. However, many microbes cannot be cultivated simply because their niches are too specific. As a result of that, in any given sample there is always a number of impossible-to-cultivate microbes that will not be detected. On the other hand, shotgun metagenomic approaches are also prone to errors because they identify any sequences that are present in the samples regardless of whether they come from microbes that are present in the sample or sequences from dead microbes. Despite how the tendency to miscalculate gene diversity of the two approaches is in opposite directions, it has always been assumed that their results (when examining the same samples) should largely be in agreement. Fifteen of the samples that we used, originating from composite samples of feces, had been used for shotgun metagenomics sequencing [26] and for isolating *Escherichia* strains and independent genome sequencing [23]. Therefore, we took advantage of that unique opportunity to test the above hypothesis. Using the metagenomic reads from the fifteen samples as well as the genome sequences from the 150 *Escherichia* isolates (for each fecal sample ten *Escherichia* isolates were genome sequenced; [23]), we mapped the predicted ORFs from both datasets to the entire VFdb database (not the pathogenic subset we used in the manuscript but the entire dataset). This showed that in the fifteen independent comparisons an average of 76% (ranging from 38% to 89%) of the *Escherichia* annotations were identical to the ones from genome sequences coming from the *Escherichia* isolates. In the venn diagrams presented here, the light green circle represents the genes coming from *Escherichia* isolates (culture-based approach) and the purple circle illustrate the genes identified using the shotgun metagenomic approach (metaG). The numbers inside the circles show the unique number of annotations to each group as well as the common. At the bottom right of each Venn diagram, the percentage (inside the parentheses) shows the count of common annoatation/total *Escherichia* annotations present in the metagenome (i.e. the *Escherichia* annotation that can be found in the metagenome and originate from *Escherichia* strains that could also be isolated).

## Data Availability

We have deposited the raw reads of the 70 samples from Theix, sequenced in this study, at NCBI under Bioproject accession number PRJNA681986. The fasta files containing the sequences of the high-quality 2114 MAGs are available at 10.15454/UIJTJA and a detailed description at https://www.fecobiome.com/resources/reference-genomes/.
