# Supplementary material for: A global phylogenomic and metabolic reconstruction of the large intestine bacterial community of domesticated cattle
Source: Microbiome. 2022 Sep 26;10:155. doi: 10.1186/s40168-022-01357-1 (PMC9511753; doi:10.1186/s40168-022-01357-1)

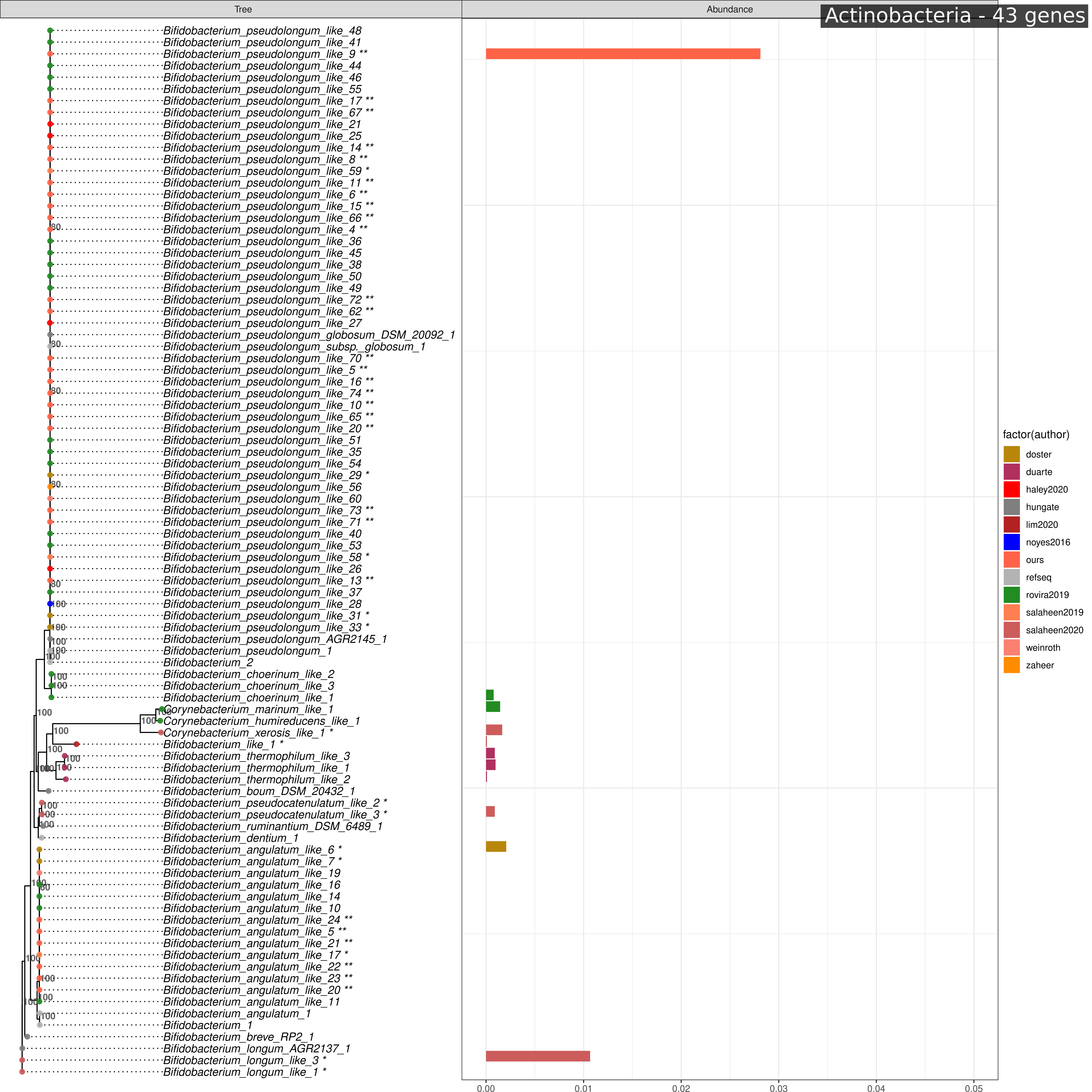
Actinobacteria - 43 genes
Tree
Abundance
Bifidobacterium_pseudolongum_like_48
Bifidobacterium_pseudolongum_like_41
Bifidobacterium_pseudolongum_like_9 **
Bifidobacterium_pseudolongum_like_44
Bifidobacterium_pseudolongum_like_46
Bifidobacterium_pseudolongum_like_55
Bifidobacterium_pseudolongum_like_17 **
Bifidobacterium_pseudolongum_like_67 **
Bifidobacterium_pseudolongum_like_21
Bifidobacterium_pseudolongum_like_25
Bifidobacterium_pseudolongum_like_14 **
Bifidobacterium_pseudolongum_like_8 **
Bifidobacterium_pseudolongum_like_59 *
Bifidobacterium_pseudolongum_like_11 **
Bifidobacterium_pseudolongum_like_6 **
Bifidobacterium_pseudolongum_like_15 **
Bifidobacterium_pseudolongum_like_66 **
Bifidobacterium_pseudolongum_like_4 **
Bifidobacterium_pseudolongum_like_36
Bifidobacterium_pseudolongum_like_45
Bifidobacterium_pseudolongum_like_38
Bifidobacterium_pseudolongum_like_50
Bifidobacterium_pseudolongum_like_49
Bifidobacterium_pseudolongum_like_72 **
Bifidobacterium_pseudolongum_like_62 **
Bifidobacterium_pseudolongum_like_27
Bifidobacterium_pseudolongum_globosum_DSM_20092_1
Bifidobacterium_pseudolongum_subsp._globosum_1
Bifidobacterium_pseudolongum_like_70 **
Bifidobacterium_pseudolongum_like_5 **
Bifidobacterium_pseudolongum_like_16 **
Bifidobacterium_pseudolongum_like_74 **
Bifidobacterium_pseudolongum_like_10 **
Bifidobacterium_pseudolongum_like_65 **
Bifidobacterium_pseudolongum_like_20 **
Bifidobacterium_pseudolongum_like_51
Bifidobacterium_pseudolongum_like_35
Bifidobacterium_pseudolongum_like_54
Bifidobacterium_pseudolongum_like_29 *
Bifidobacterium_pseudolongum_like_56
Bifidobacterium_pseudolongum_like_60
Bifidobacterium_pseudolongum_like_73 **
Bifidobacterium_pseudolongum_like_71 **
Bifidobacterium_pseudolongum_like_40
Bifidobacterium_pseudolongum_like_53
Bifidobacterium_pseudolongum_like_58 *
Bifidobacterium_pseudolongum_like_26
Bifidobacterium_pseudolongum_like_13 **
Bifidobacterium_pseudolongum_like_37
Bifidobacterium_pseudolongum_like_28
Bifidobacterium_pseudolongum_like_31 *
Bifidobacterium_pseudolongum_like_33 *
Bifidobacterium_pseudolongum_AGR2145_1
Bifidobacterium_pseudolongum_1
Bifidobacterium_2
Bifidobacterium_choerinum_like_2
Bifidobacterium_choerinum_like_3
Bifidobacterium_choerinum_like_1
Corynebacterium_marinum_like_1
Corynebacterium_humireducens_like_1
Corynebacterium_xerosis_like_1 *
Bifidobacterium_like_1 *
Bifidobacterium_thermophilum_like_3
Bifidobacterium_thermophilum_like_1
Bifidobacterium_thermophilum_like_2
Bifidobacterium_boum_DSM_20432_1
Bifidobacterium_pseudocatenulatum_like_2 *
Bifidobacterium_pseudocatenulatum_like_3 *
Bifidobacterium_ruminantium_DSM_6489_1
Bifidobacterium_dentium_1
Bifidobacterium_angulatum_like_6 *
Bifidobacterium_angulatum_like_7 *
Bifidobacterium_angulatum_like_19
Bifidobacterium_angulatum_like_16
Bifidobacterium_angulatum_like_14
Bifidobacterium_angulatum_like_10
Bifidobacterium_angulatum_like_24 **
Bifidobacterium_angulatum_like_5 **
Bifidobacterium_angulatum_like_21 **
Bifidobacterium_angulatum_like_17 *
Bifidobacterium_angulatum_like_22 **
Bifidobacterium_angulatum_like_23 **
Bifidobacterium_angulatum_like_20 **
Bifidobacterium_angulatum_like_11
Bifidobacterium_angulatum_1
Bifidobacterium_1
Bifidobacterium_breve_RP2_1
Bifidobacterium_longum_AGR2137_1
Bifidobacterium_longum_like_3 *
Bifidobacterium_longum_like_1 *
0.00
0.01
0.02
0.03
0.04
0.05
factor(author)
doster
duarte
haley2020
hungate
lim2020
noyes2016
ours
refseq
rovira2019
salaheen2019
salaheen2020
weinroth
zaheer

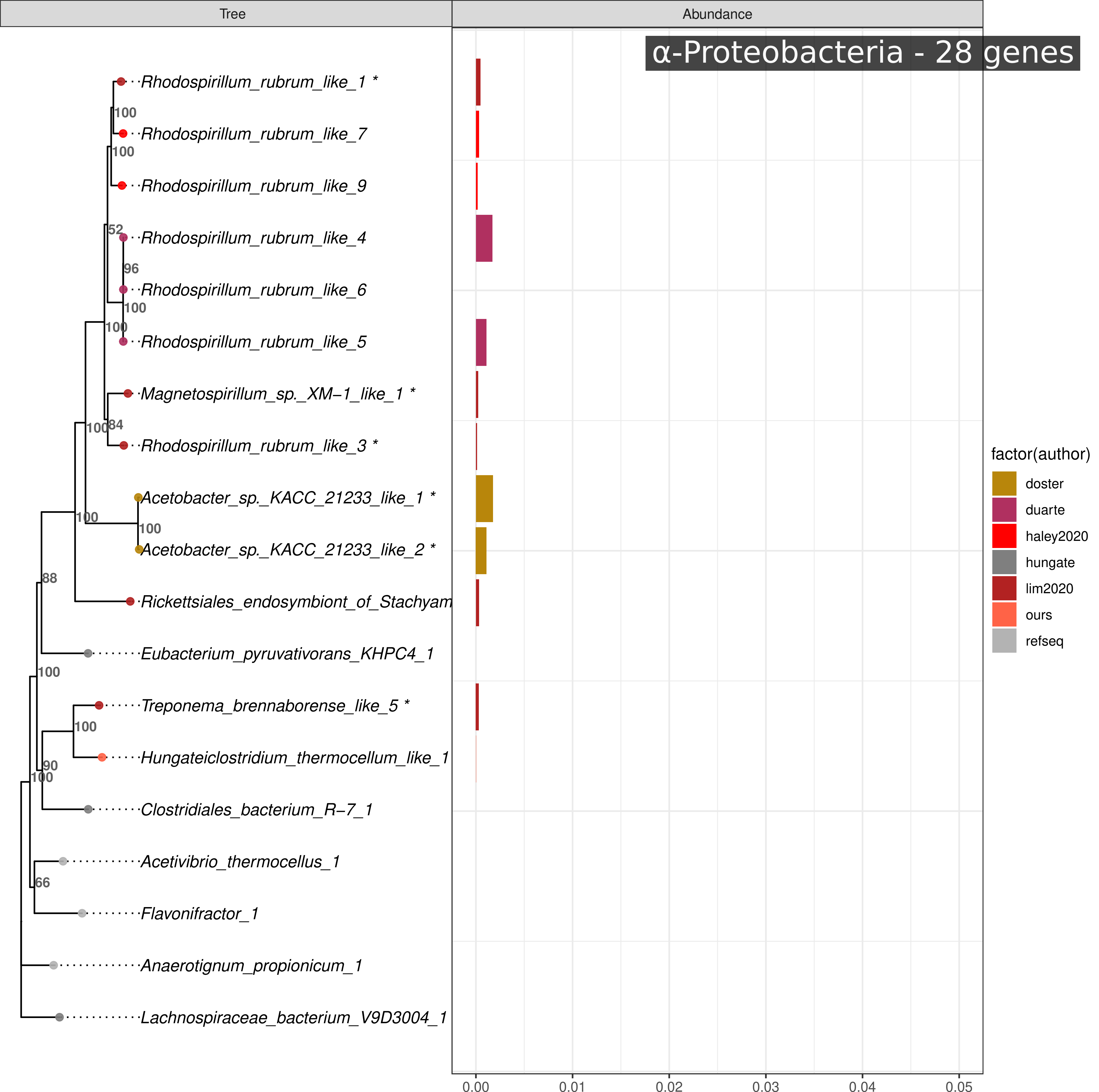

Tree
Abundance
α-Proteobacteria - 28 genes
Rhodospirillum_rubrum_like_1 *
Rhodospirillum_rubrum_like_7
Rhodospirillum_rubrum_like_9
Rhodospirillum_rubrum_like_4
Rhodospirillum_rubrum_like_6
Rhodospirillum_rubrum_like_5
Magnetospirillum_sp._XM−1_like_1 *
Rhodospirillum_rubrum_like_3 *
Acetobacter_sp._KACC_21233_like_1 *
Acetobacter_sp._KACC_21233_like_2 *
Rickettsiales_endosymbiont_of_Stachyam
Eubacterium_pyruvativorans_KHPC4_1
Treponema_brennaborense_like_5 *
Hungateiclostridium_thermocellum_like_1
Clostridiales_bacterium_R−7_1
Acetivibrio_thermocellus_1
Flavonifractor_1
Anaerotignum_propionicum_1
Lachnospiraceae_bacterium_V9D3004_1
100
100
52
96
100
100
100
84
100
100
88
100
100
90
100
66
0.00
0.01
0.02
0.03
0.04
0.05
factor(author)
doster
duarte
haley2020
hungate
lim2020
ours
refseq

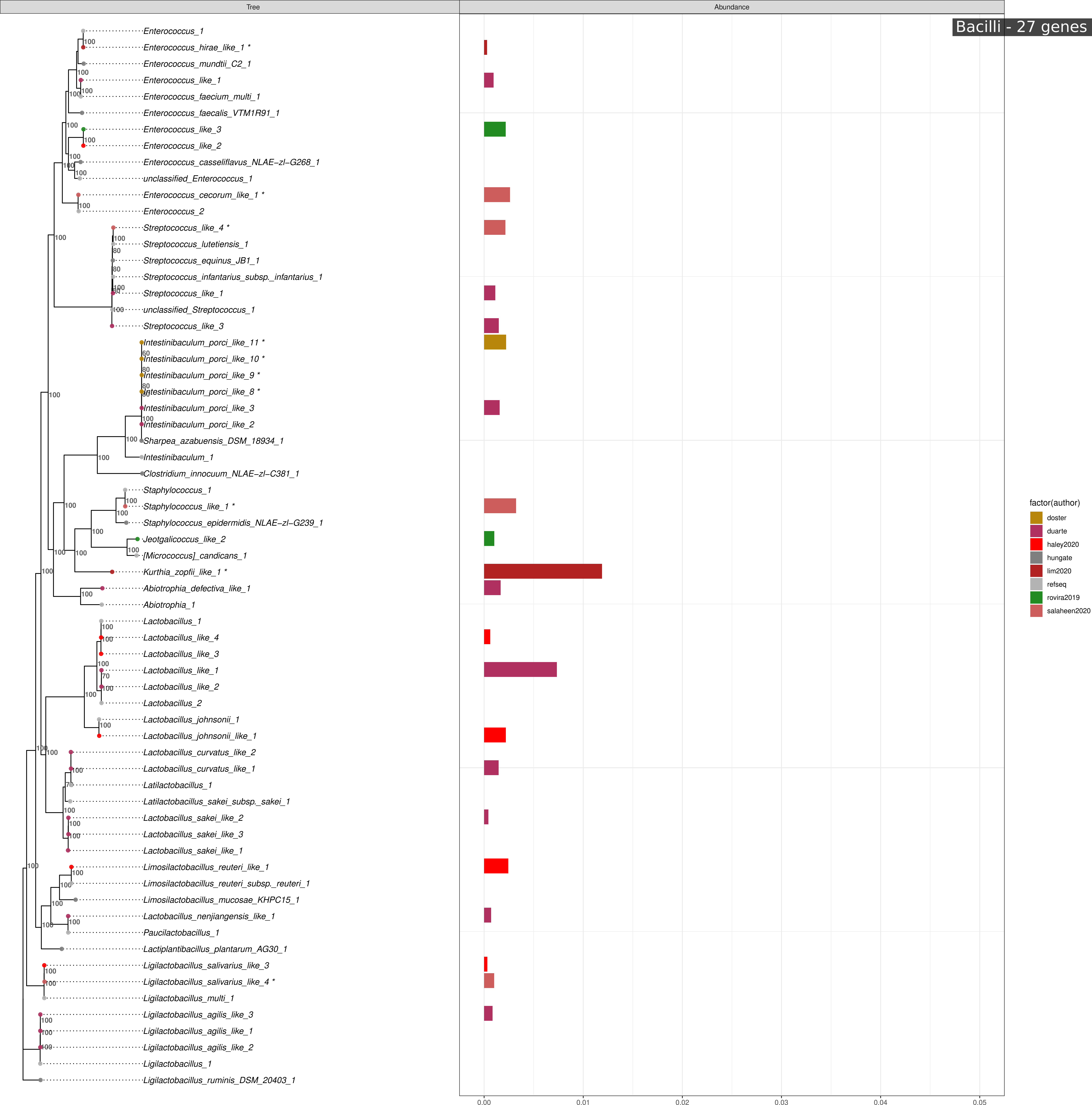

Tree
Abundance
Bacilli - 27 genes
Enterococcus_1
Enterococcus_hirae_like_1 *
Enterococcus_mundtii_C2_1
Enterococcus_like_1
Enterococcus_faecium_multi_1
Enterococcus_faecalis_VTM1R91_1
Enterococcus_like_3
Enterococcus_like_2
Enterococcus_casseliflavus_NLAE-zl-G268_1
unclassified_Enterococcus_1
Enterococcus_cecorum_like_1 *
Enterococcus_2
Streptococcus_like_4 *
Streptococcus_lutetiensis_1
Streptococcus_equinus_JB1_1
Streptococcus_infantarius_subsp._infantarius_1
Streptococcus_like_1
unclassified_Streptococcus_1
Streptococcus_like_3
Intestinibaculum_porci_like_11 *
Intestinibaculum_porci_like_10 *
Intestinibaculum_porci_like_9 *
Intestinibaculum_porci_like_8 *
Intestinibaculum_porci_like_3
Intestinibaculum_porci_like_2
Sharpea_azabuensis_DSM_18934_1
Intestinibaculum_1
Clostridium_innocuum_NLAE-zl-C381_1
Staphylococcus_1
Staphylococcus_like_1 *
Staphylococcus_epidermidis_NLAE-zl-G239_1
Jeotgalicoccus_like_2
[Micrococcus]_candicans_1
Kurthia_zopfii_like_1 *
Abiotrophia_defectiva_like_1
Abiotrophia_1
Lactobacillus_1
Lactobacillus_like_4
Lactobacillus_like_3
Lactobacillus_like_1
Lactobacillus_like_2
Lactobacillus_2
Lactobacillus_johnsonii_1
Lactobacillus_johnsonii_like_1
Lactobacillus_curvatus_like_2
Lactobacillus_curvatus_like_1
Latilactobacillus_1
Latilactobacillus_sakei_subsp._sakei_1
Lactobacillus_sakei_like_2
Lactobacillus_sakei_like_3
Lactobacillus_sakei_like_1
Limosilactobacillus_reuteri_like_1
Limosilactobacillus_reuteri_subsp._reuteri_1
Limosilactobacillus_mucosae_KHPC15_1
Lactobacillus_nenjiangensis_like_1
Paucilactobacillus_1
Lactiplantibacillus_plantarum_AG30_1
Ligilactobacillus_salivarius_like_3
Ligilactobacillus_salivarius_like_4 *
Ligilactobacillus_multi_1
Ligilactobacillus_agilis_like_3
Ligilactobacillus_agilis_like_1
Ligilactobacillus_agilis_like_2
Ligilactobacillus_1
Ligilactobacillus_ruminis_DSM_20403_1
factor(author)
doster
duarte
haley2020
hungate
lim2020
refseq
rovira2019
salaheen2020
0.00
0.01
0.02
0.03
0.04
0.05

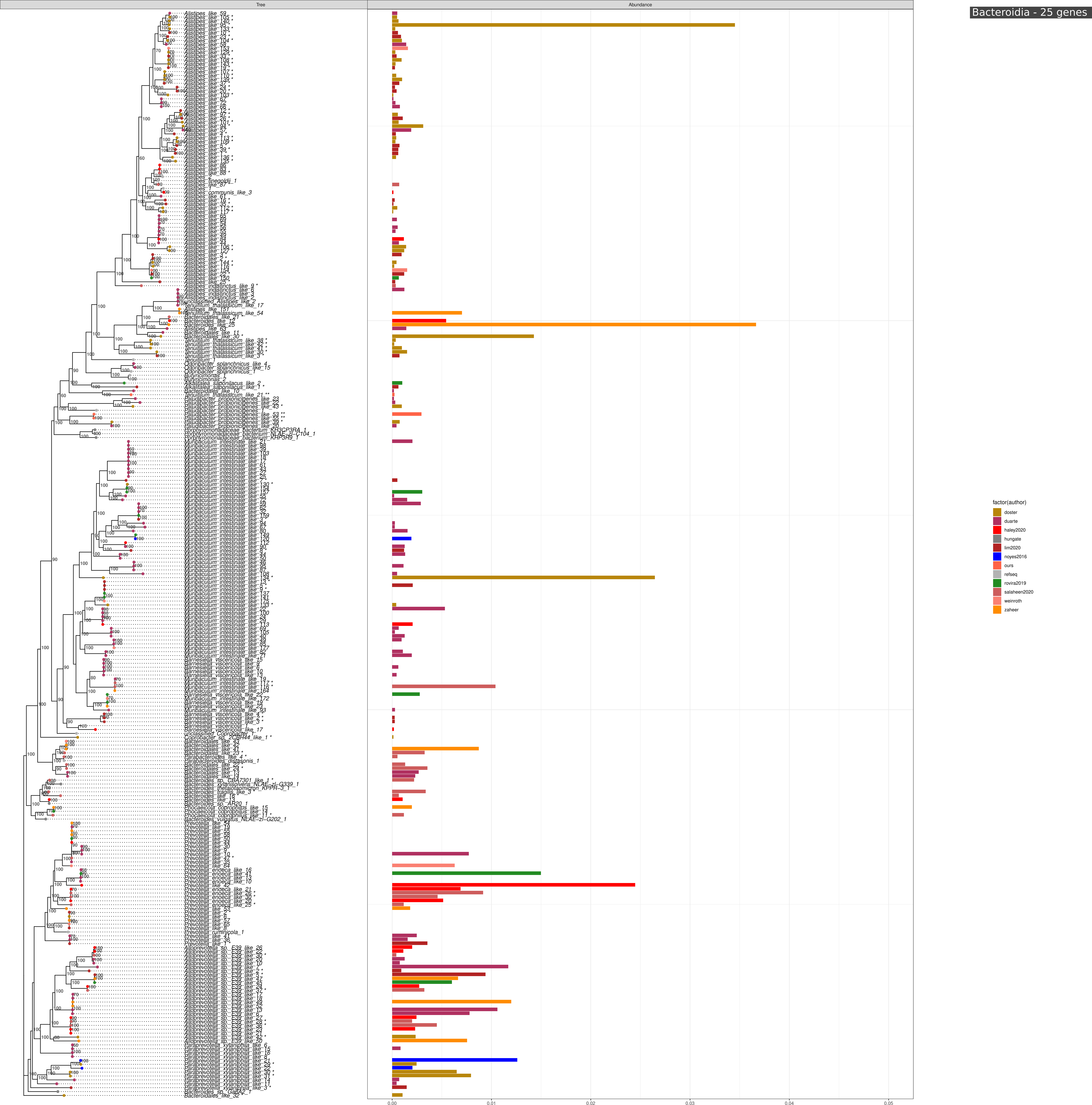

Bacteroidia - 25 genes
Tree
Abundance
0.00
0.01
0.02
0.03
0.04
0.05
factor(author)
doster
duarte
haley2020
hungate
lim2020
noyes2016
ours
refseq
rovira2019
salaheen2020
weinroth
zaheer

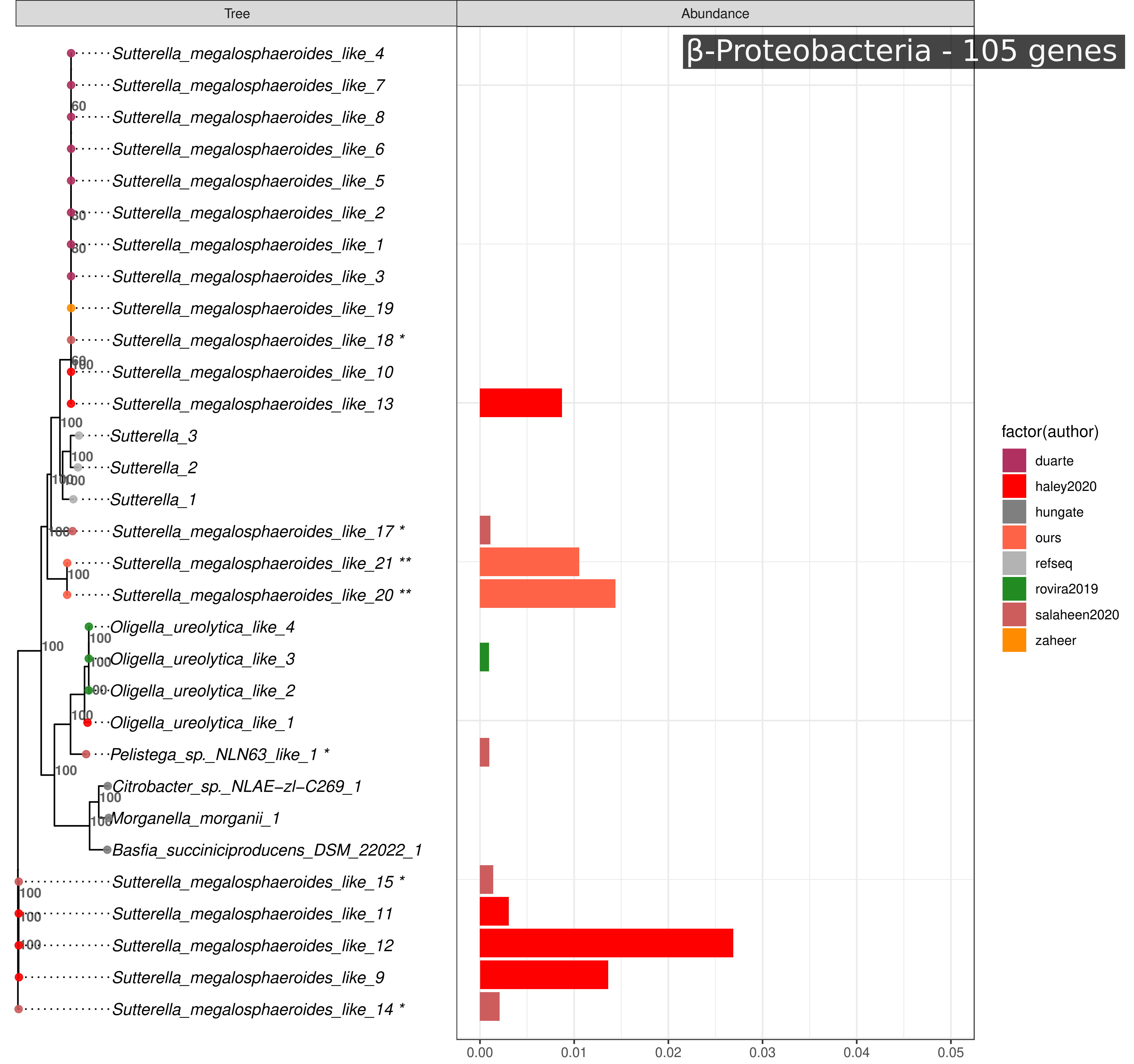

Tree
Abundance
β-Proteobacteria - 105 genes
Sutterella_megalosphaeroides_like_4
Sutterella_megalosphaeroides_like_7
Sutterella_megalosphaeroides_like_8
Sutterella_megalosphaeroides_like_6
Sutterella_megalosphaeroides_like_5
Sutterella_megalosphaeroides_like_2
Sutterella_megalosphaeroides_like_1
Sutterella_megalosphaeroides_like_3
Sutterella_megalosphaeroides_like_19
Sutterella_megalosphaeroides_like_18 *
Sutterella_megalosphaeroides_like_10
Sutterella_megalosphaeroides_like_13
Sutterella_3
Sutterella_2
Sutterella_1
Sutterella_megalosphaeroides_like_17 *
Sutterella_megalosphaeroides_like_21 **
Sutterella_megalosphaeroides_like_20 **
Oligella_ureolytica_like_4
Oligella_ureolytica_like_3
Oligella_ureolytica_like_2
Oligella_ureolytica_like_1
Pelistega_sp._NLN63_like_1 *
Citrobacter_sp._NLAE-zl-C269_1
Morganella_morganii_1
Basfia_succiniciproducens_DSM_22022_1
Sutterella_megalosphaeroides_like_15 *
Sutterella_megalosphaeroides_like_11
Sutterella_megalosphaeroides_like_12
Sutterella_megalosphaeroides_like_9
Sutterella_megalosphaeroides_like_14 *
0.00
0.01
0.02
0.03
0.04
0.05
factor(author)
duarte
haley2020
hungate
ours
refseq
rovira2019
salaheen2020
zaheer

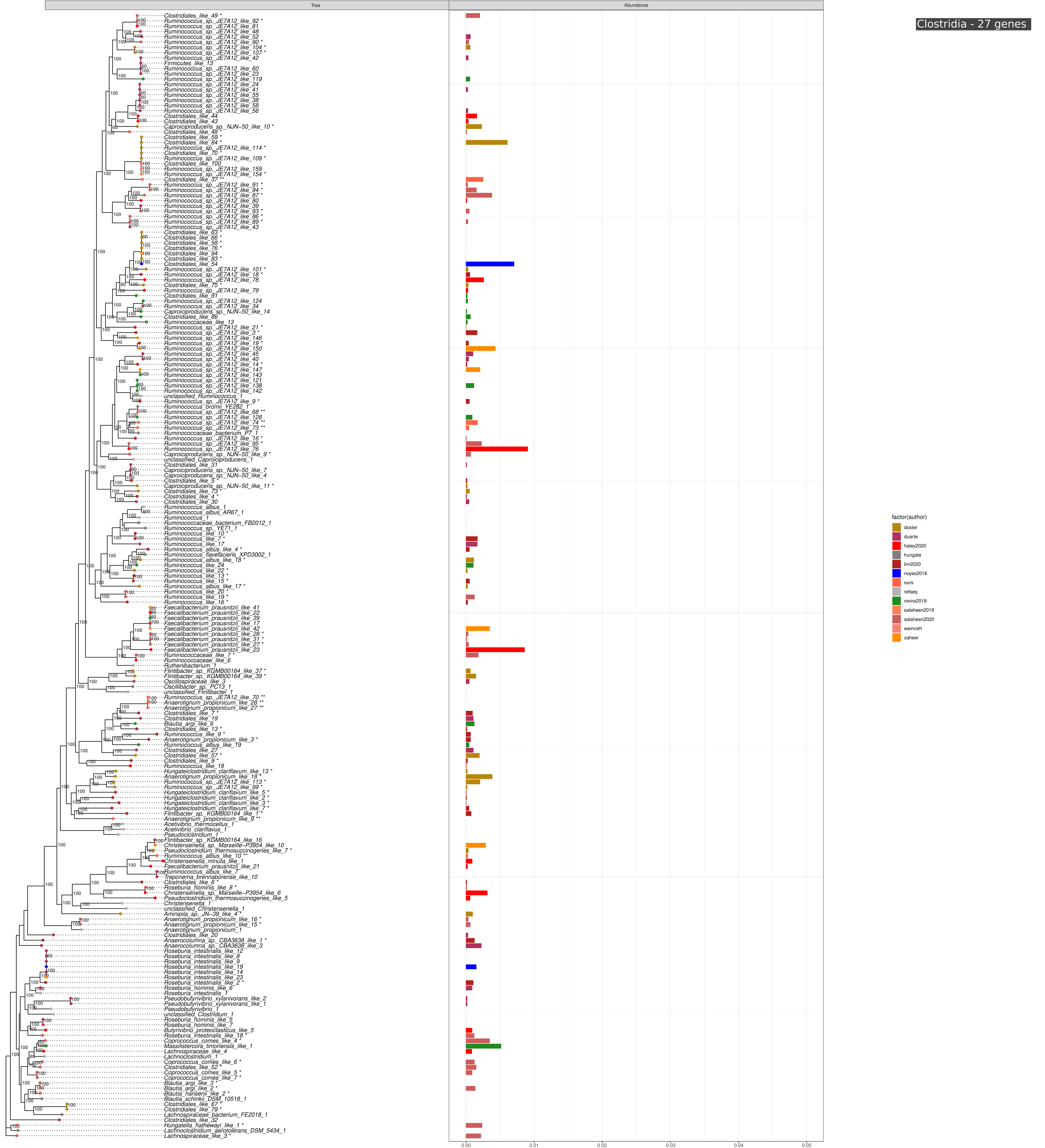
Tree
Abundance
Clostridia - 27 genes
Clostridiales_like_49 *
Ruminococcus_sp._JE7A12_like_92 *
Ruminococcus_sp._JE7A12_like_81
Ruminococcus_sp._JE7A12_like_48
Ruminococcus_sp._JE7A12_like_52
Ruminococcus_sp._JE7A12_like_90 *
Ruminococcus_sp._JE7A12_like_104 *
Ruminococcus_sp._JE7A12_like_107 *
Ruminococcus_sp._JE7A12_like_42
Firmicutes_like_13
Ruminococcus_sp._JE7A12_like_60
Ruminococcus_sp._JE7A12_like_23
Ruminococcus_sp._JE7A12_like_119
Ruminococcus_sp._JE7A12_like_24
Ruminococcus_sp._JE7A12_like_41
Ruminococcus_sp._JE7A12_like_55
Ruminococcus_sp._JE7A12_like_38
Ruminococcus_sp._JE7A12_like_58
Ruminococcus_sp._JE7A12_like_56
Clostridiales_like_44
Clostridiales_like_43
Caproiciproducens_sp._NJN−50_like_10 *
Clostridiales_like_48 *
Clostridiales_like_59 *
Clostridiales_like_84 *
Ruminococcus_sp._JE7A12_like_114 *
Clostridiales_like_70 *
Ruminococcus_sp._JE7A12_like_109 *
Clostridiales_like_100
Ruminococcus_sp._JE7A12_like_159
Ruminococcus_sp._JE7A12_like_154 *
Clostridiales_like_37 **
Ruminococcus_sp._JE7A12_like_91 *
Ruminococcus_sp._JE7A12_like_94 *
Ruminococcus_sp._JE7A12_like_87 *
Ruminococcus_sp._JE7A12_like_80
Ruminococcus_sp._JE7A12_like_39
Ruminococcus_sp._JE7A12_like_93 *
Ruminococcus_sp._JE7A12_like_86 *
Ruminococcus_sp._JE7A12_like_89 *
Ruminococcus_sp._JE7A12_like_43
Clostridiales_like_63 *
Clostridiales_like_66 *
Clostridiales_like_58 *
Clostridiales_like_76 *
Clostridiales_like_94
Clostridiales_like_83 *
Clostridiales_like_54
Ruminococcus_sp._JE7A12_like_101 *
Ruminococcus_sp._JE7A12_like_18 *
Ruminococcus_sp._JE7A12_like_78
Clostridiales_like_75 *
Ruminococcus_sp._JE7A12_like_79
Clostridiales_like_91
Ruminococcus_sp._JE7A12_like_124
Ruminococcus_sp._JE7A12_like_34
Caproiciproducens_sp._NJN−50_like_14
Clostridiales_like_86
Ruminococcaceae_like_13
Ruminococcus_sp._JE7A12_like_21 *
Ruminococcus_sp._JE7A12_like_3 *
Ruminococcus_sp._JE7A12_like_146
Ruminococcus_sp._JE7A12_like_19 *
Ruminococcus_sp._JE7A12_like_150
Ruminococcus_sp._JE7A12_like_45
Ruminococcus_sp._JE7A12_like_40
Ruminococcus_sp._JE7A12_like_14 *
Ruminococcus_sp._JE7A12_like_147
Ruminococcus_sp._JE7A12_like_143
Ruminococcus_sp._JE7A12_like_121
Ruminococcus_sp._JE7A12_like_138
Ruminococcus_sp._JE7A12_like_142
unclassified_Ruminococcus_1
Ruminococcus_sp._JE7A12_like_9 *
Ruminococcus_bromii_YE282_1
Ruminococcus_sp._JE7A12_like_68 **
Ruminococcus_sp._JE7A12_like_128
Ruminococcus_sp._JE7A12_like_74 **
Ruminococcus_sp._JE7A12_like_73 **
Ruminococcaceae_bacterium_P7_1
Ruminococcus_sp._JE7A12_like_16 *
Ruminococcus_sp._JE7A12_like_95 *
Ruminococcus_sp._JE7A12_like_76
Caproiciproducens_sp._NJN−50_like_9 *
unclassified_Caproiciproducens_1
Clostridiales_like_31
Caproiciproducens_sp._NJN−50_like_7
Caproiciproducens_sp._NJN−50_like_4
Clostridiales_like_5 *
Caproiciproducens_sp._NJN−50_like_11 *
Clostridiales_like_73 *
Clostridiales_like_4 *
Clostridiales_like_30
Ruminococcus_albus_1
Ruminococcus_albus_AR67_1
Ruminococcus_1
Ruminococcaceae_bacterium_FB2012_1
Ruminococcus_sp._YE71_1
Ruminococcus_like_10 *
Ruminococcus_like_7 *
Ruminococcus_like_17
Ruminococcus_albus_like_4 *
Ruminococcus_flavefaciens_XPD3002_1
Ruminococcus_albus_like_18 *
Ruminococcus_like_24
Ruminococcus_like_22 *
Ruminococcus_like_13 *
Ruminococcus_like_15 *
Ruminococcus_albus_like_17 *
Ruminococcus_like_20 *
Ruminococcus_like_19 *
Ruminococcus_like_16 *
Faecalibacterium_prausnitzii_like_41
Faecalibacterium_prausnitzii_like_22
Faecalibacterium_prausnitzii_like_39
Faecalibacterium_prausnitzii_like_17
Faecalibacterium_prausnitzii_like_42
Faecalibacterium_prausnitzii_like_28 *
Faecalibacterium_prausnitzii_like_31 *
Faecalibacterium_prausnitzii_like_27 *
Faecalibacterium_prausnitzii_like_23
Ruminococcaceae_like_7 *
Ruminococcaceae_like_6
Ruthenibacterium_1
Flintibacter_sp._KGMB00164_like_37 *
Flintibacter_sp._KGMB00164_like_39 *
Oscillospiraceae_like_3
Oscillibacter_sp._PC13_1
unclassified_Flintibacter_1
Ruminococcus_sp._JE7A12_like_70 **
Anaerotignum_propionicum_like_28 **
Anaerotignum_propionicum_like_27 **
Clostridiales_like_7 *
Clostridiales_like_19
Blautia_argi_like_6
Clostridiales_like_13 *
Ruminococcus_like_9 *
Anaerotignum_propionicum_like_3 *
Ruminococcus_albus_like_19
Clostridiales_like_27
Clostridiales_like_57 *
Clostridiales_like_9 *
Ruminococcus_like_18
Hungateiclostridium_clariflavum_like_13 *
Anaerotignum_propionicum_like_18 *
Ruminococcus_sp._JE7A12_like_113 *
Ruminococcus_sp._JE7A12_like_99 *
Hungateiclostridium_clariflavum_like_5 *
Hungateiclostridium_clariflavum_like_2 *
Hungateiclostridium_clariflavum_like_3 *
Hungateiclostridium_clariflavum_like_7 *
Flintibacter_sp._KGMB00164_like_1 *
Anaerotignum_propionicum_like_9 **
Acetivibrio_thermocellus_1
Acetivibrio_clariflavus_1
Pseudoclostridium_1
Flintibacter_sp._KGMB00164_like_16
Christensenella_sp._Marseille−P3954_like_10
Pseudoclostridium_thermosuccinogenes_like_7 *
Ruminococcus_albus_like_10 **
Christensenella_minuta_like_1
Faecalibacterium_prausnitzii_like_21
Ruminococcus_albus_like_7
Treponema_brennaborense_like_10
Clostridiales_like_6 *
Roseburia_hominis_like_8 *
Christensenella_sp._Marseille−P3954_like_6
Pseudoclostridium_thermosuccinogenes_like_5
Christensenella_1
unclassified_Christensenella_1
Aminipila_sp._JN−39_like_4 *
Anaerotignum_propionicum_like_16 *
Anaerotignum_propionicum_like_15 *
Anaerotignum_propionicum_1
Clostridiales_like_20
Anaerocolumna_sp._CBA3638_like_1 *
Anaerocolumna_sp._CBA3638_like_3
Roseburia_intestinalis_like_12
Roseburia_intestinalis_like_8
Roseburia_intestinalis_like_9
Roseburia_intestinalis_like_19
Roseburia_intestinalis_like_14
Roseburia_intestinalis_like_23
Roseburia_intestinalis_like_2 *
Roseburia_hominis_like_6
Roseburia_intestinalis_1
Pseudobutyrivibrio_xylanivorans_like_2
Pseudobutyrivibrio_xylanivorans_like_1
Pseudobutyrivibrio_1
unclassified_Clostridium_1
Roseburia_hominis_like_5
Roseburia_hominis_like_7
Butyrivibrio_proteoclasticus_like_5
Roseburia_intestinalis_like_18 *
Coprococcus_comes_like_4 *
Massilistercora_timonensis_like_1
Lachnospiraceae_like_4
Lachnoclostridium_1
Coprococcus_comes_like_6 *
Clostridiales_like_52 *
Coprococcus_comes_like_5 *
Coprococcus_comes_like_7 *
Blautia_argi_like_3 *
Blautia_argi_like_2 *
Blautia_hansenii_like_2 *
Blautia_schinkii_DSM_10518_1
Clostridiales_like_67 *
Clostridiales_like_79 *
Lachnospiraceae_bacterium_FE2018_1
Clostridiales_like_32
Hungatella_hathewayi_like_1 *
Lachnoclostridium_aerotolerans_DSM_5434_1
Lachnospiraceae_like_3 *
0.00
0.01
0.02
0.03
0.04
0.05
factor(author)
doster
duarte
haley2020
hungate
lim2020
noyes2016
ours
refseq
rovira2019
salaheen2019
salaheen2020
weinroth
zaheer

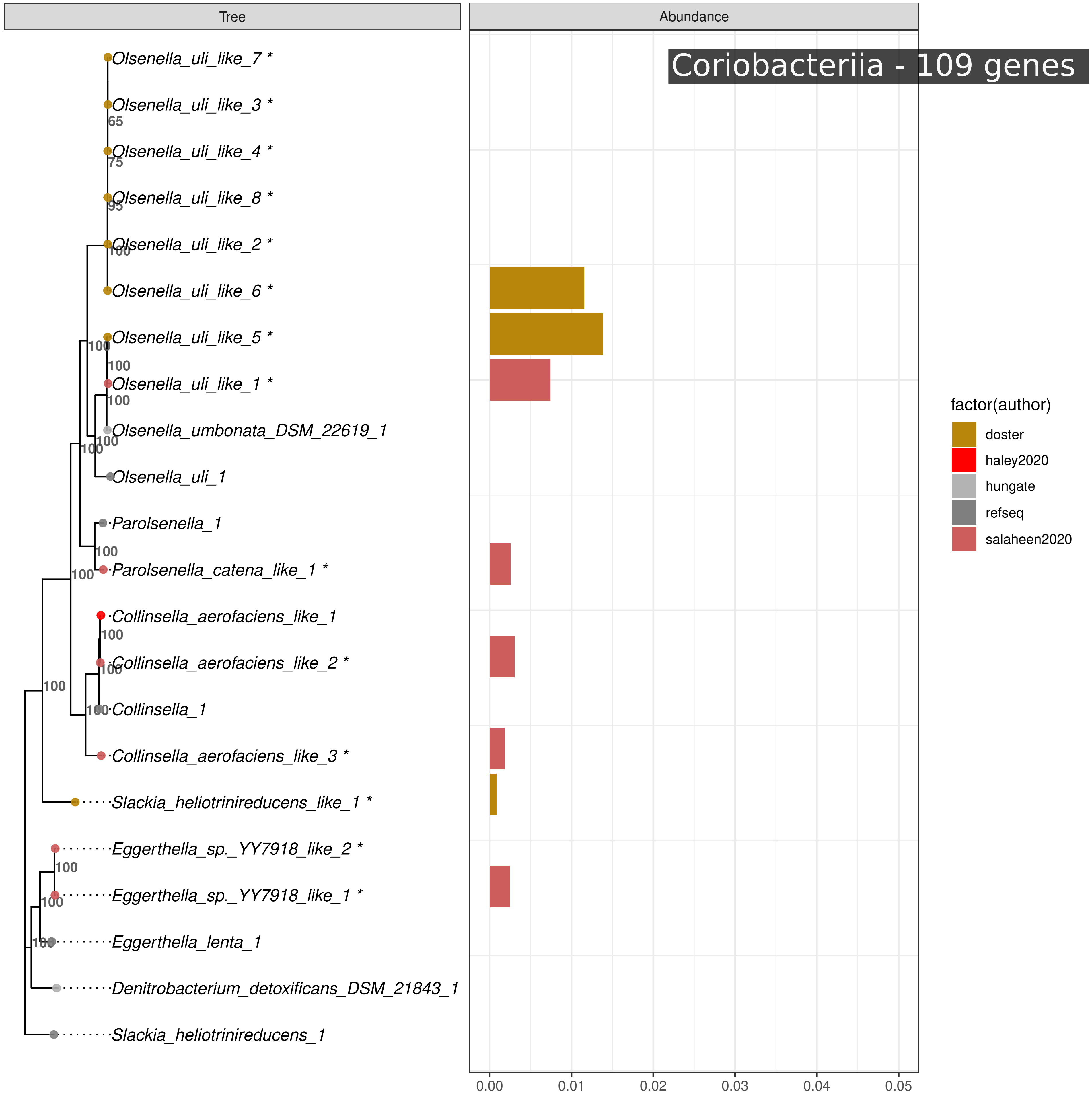

Tree
Abundance
Coriobacteriia - 109 genes
Olsenella_uli_like_7 *
Olsenella_uli_like_3 *
Olsenella_uli_like_4 *
Olsenella_uli_like_8 *
Olsenella_uli_like_2 *
Olsenella_uli_like_6 *
Olsenella_uli_like_5 *
Olsenella_uli_like_1 *
Olsenella_umbonata_DSM_22619_1
Olsenella_uli_1
Parolsenella_1
Parolsenella_catena_like_1 *
Collinsella_aerofaciens_like_1
Collinsella_aerofaciens_like_2 *
Collinsella_1
Collinsella_aerofaciens_like_3 *
Slackia_heliotrinireducens_like_1 *
Eggerthella_sp._YY7918_like_2 *
Eggerthella_sp._YY7918_like_1 *
Eggerthella_lenta_1
Denitrobacterium_detoxificans_DSM_21843_1
Slackia_heliotrinireducens_1
65
75
95
100
0.00
0.01
0.02
0.03
0.04
0.05
factor(author)
doster
haley2020
hungate
refseq
salaheen2020

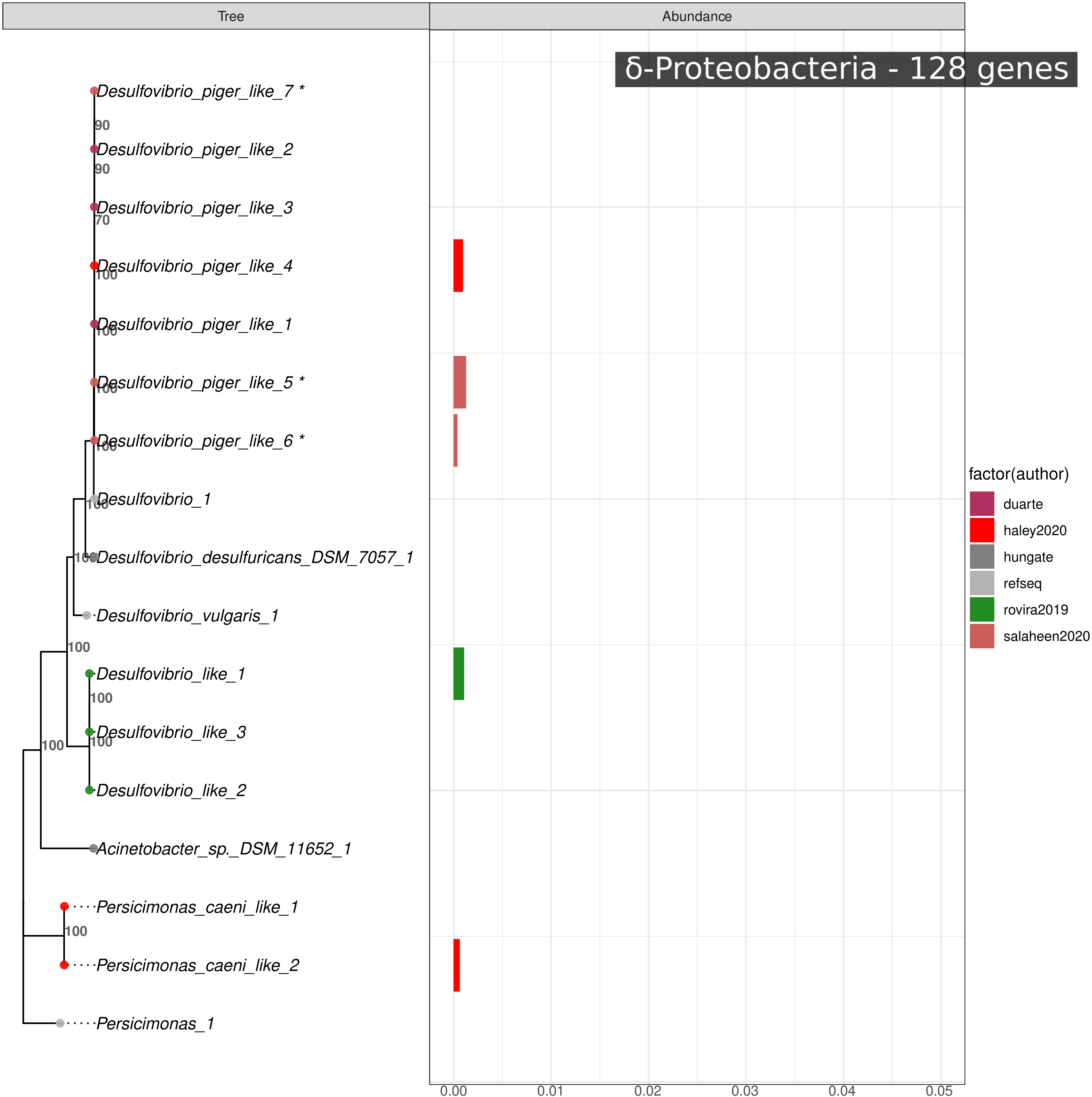

Tree
Abundance
δ-Proteobacteria - 128 genes
Desulfovibrio_piger_like_7 *
Desulfovibrio_piger_like_2
Desulfovibrio_piger_like_3
Desulfovibrio_piger_like_4
Desulfovibrio_piger_like_1
Desulfovibrio_piger_like_5 *
Desulfovibrio_piger_like_6 *
Desulfovibrio_1
Desulfovibrio_desulfuricans_DSM_7057_1
Desulfovibrio_vulgaris_1
Desulfovibrio_like_1
Desulfovibrio_like_3
Desulfovibrio_like_2
Acinetobacter_sp._DSM_11652_1
Persicimonas_caeni_like_1
Persicimonas_caeni_like_2
Persicimonas_1
90
90
70
100
100
100
100
100
100
100
100
100
100
0.00
0.01
0.02
0.03
0.04
0.05
factor(author)
duarte
haley2020
hungate
refseq
rovira2019
salaheen2020

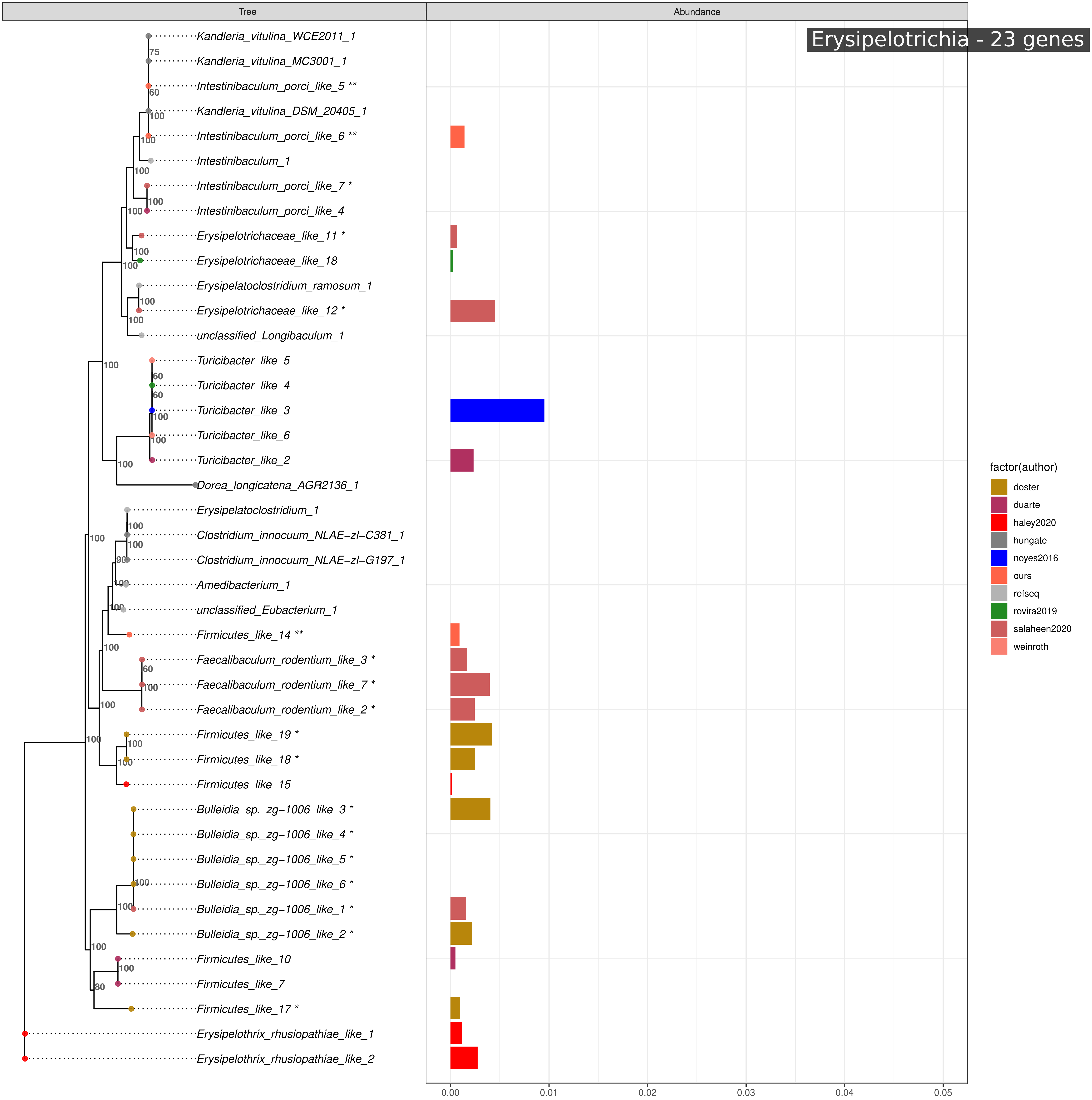
Tree
Abundance
Erysipelotrichia - 23 genes
Kandleria_vitulina_WCE2011_1
Kandleria_vitulina_MC3001_1
Intestinibaculum_porci_like_5 **
Kandleria_vitulina_DSM_20405_1
Intestinibaculum_porci_like_6 **
Intestinibaculum_1
Intestinibaculum_porci_like_7 *
Intestinibaculum_porci_like_4
Erysipelotrichaceae_like_11 *
Erysipelotrichaceae_like_18
Erysipelatoclostridium_ramosum_1
Erysipelotrichaceae_like_12 *
unclassified_Longibaculum_1
Turicibacter_like_5
Turicibacter_like_4
Turicibacter_like_3
Turicibacter_like_6
Turicibacter_like_2
Dorea_longicatena_AGR2136_1
Erysipelatoclostridium_1
Clostridium_innocuum_NLAE−zl−C381_1
Clostridium_innocuum_NLAE−zl−G197_1
Amedibacterium_1
unclassified_Eubacterium_1
Firmicutes_like_14 **
Faecalibaculum_rodentium_like_3 *
Faecalibaculum_rodentium_like_7 *
Faecalibaculum_rodentium_like_2 *
Firmicutes_like_19 *
Firmicutes_like_18 *
Firmicutes_like_15
Bulleidia_sp._zg−1006_like_3 *
Bulleidia_sp._zg−1006_like_4 *
Bulleidia_sp._zg−1006_like_5 *
Bulleidia_sp._zg−1006_like_6 *
Bulleidia_sp._zg−1006_like_1 *
Bulleidia_sp._zg−1006_like_2 *
Firmicutes_like_10
Firmicutes_like_7
Firmicutes_like_17 *
Erysipelothrix_rhusiopathiae_like_1
Erysipelothrix_rhusiopathiae_like_2
0.00
0.01
0.02
0.03
0.04
0.05
factor(author)
doster
duarte
haley2020
hungate
noyes2016
ours
refseq
rovira2019
salaheen2020
weinroth

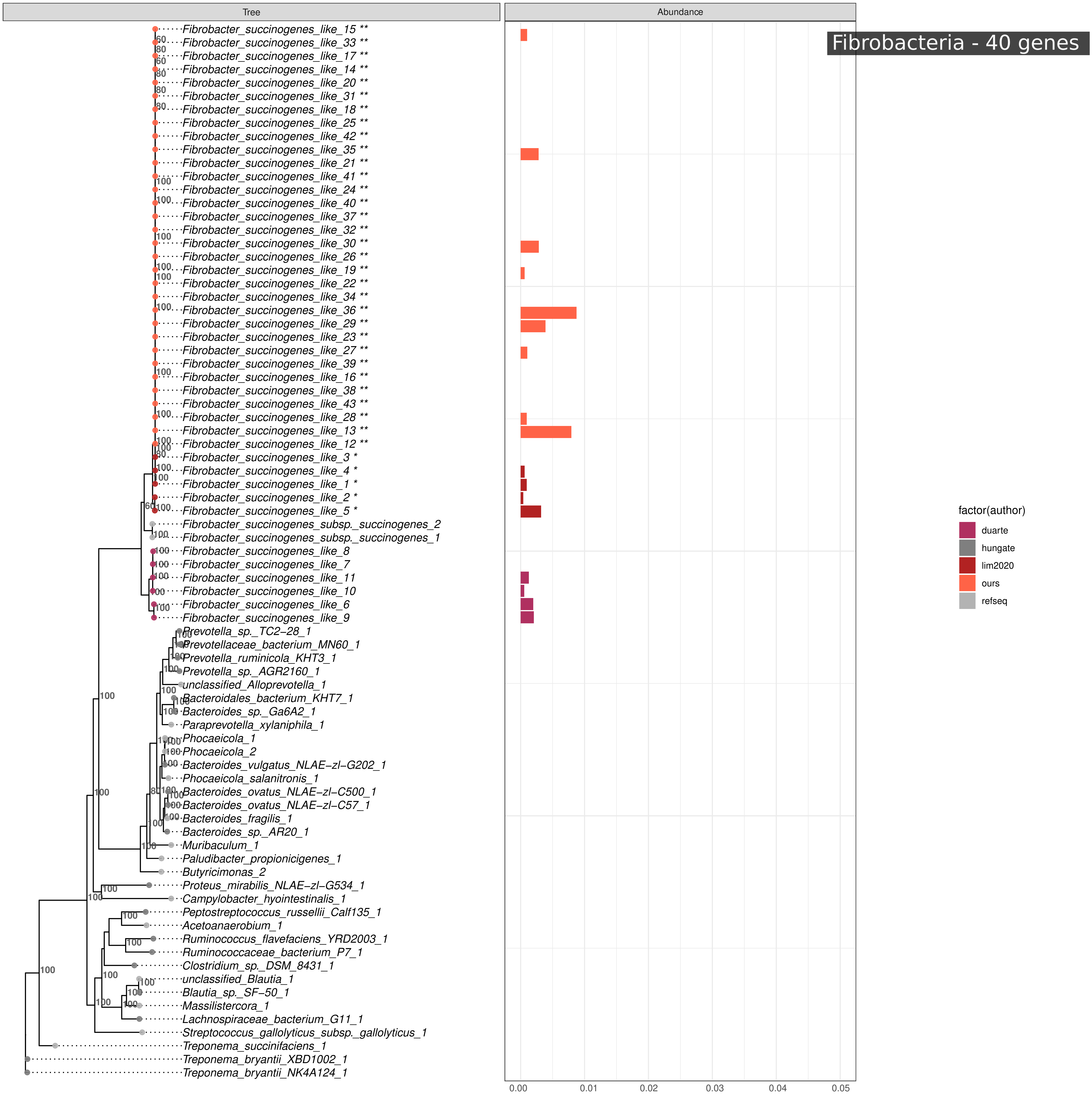
Tree
Abundance
Fibrobacteria - 40 genes
Fibrobacter_succinogenes_like_15 **
Fibrobacter_succinogenes_like_33 **
Fibrobacter_succinogenes_like_17 **
Fibrobacter_succinogenes_like_14 **
Fibrobacter_succinogenes_like_20 **
Fibrobacter_succinogenes_like_31 **
Fibrobacter_succinogenes_like_18 **
Fibrobacter_succinogenes_like_25 **
Fibrobacter_succinogenes_like_42 **
Fibrobacter_succinogenes_like_35 **
Fibrobacter_succinogenes_like_21 **
Fibrobacter_succinogenes_like_41 **
Fibrobacter_succinogenes_like_24 **
Fibrobacter_succinogenes_like_40 **
Fibrobacter_succinogenes_like_37 **
Fibrobacter_succinogenes_like_32 **
Fibrobacter_succinogenes_like_30 **
Fibrobacter_succinogenes_like_26 **
Fibrobacter_succinogenes_like_19 **
Fibrobacter_succinogenes_like_22 **
Fibrobacter_succinogenes_like_34 **
Fibrobacter_succinogenes_like_36 **
Fibrobacter_succinogenes_like_29 **
Fibrobacter_succinogenes_like_23 **
Fibrobacter_succinogenes_like_27 **
Fibrobacter_succinogenes_like_39 **
Fibrobacter_succinogenes_like_16 **
Fibrobacter_succinogenes_like_38 **
Fibrobacter_succinogenes_like_43 **
Fibrobacter_succinogenes_like_28 **
Fibrobacter_succinogenes_like_13 **
Fibrobacter_succinogenes_like_12 **
Fibrobacter_succinogenes_like_3 *
Fibrobacter_succinogenes_like_4 *
Fibrobacter_succinogenes_like_1 *
Fibrobacter_succinogenes_like_2 *
Fibrobacter_succinogenes_like_5 *
Fibrobacter_succinogenes_subsp._succinogenes_2
Fibrobacter_succinogenes_subsp._succinogenes_1
Fibrobacter_succinogenes_like_8
Fibrobacter_succinogenes_like_7
Fibrobacter_succinogenes_like_11
Fibrobacter_succinogenes_like_10
Fibrobacter_succinogenes_like_6
Fibrobacter_succinogenes_like_9
Prevotella_sp._TC2-28_1
Prevotellaceae_bacterium_MN60_1
Prevotella_ruminicola_KHT3_1
Prevotella_sp._AGR2160_1
unclassified_Alloprevotella_1
Bacteroidales_bacterium_KHT7_1
Bacteroides_sp._Ga6A2_1
Paraprevotella_xylaniphila_1
Phocaeicola_1
Phocaeicola_2
Bacteroides_vulgatus_NLAE-zl-G202_1
Phocaeicola_salanitronis_1
Bacteroides_ovatus_NLAE-zl-C500_1
Bacteroides_ovatus_NLAE-zl-C57_1
Bacteroides_fragilis_1
Bacteroides_sp._AR20_1
Muribaculum_1
Paludibacter_propionicigenes_1
Butyricimonas_2
Proteus_mirabilis_NLAE-zl-G534_1
Campylobacter_hyointestinalis_1
Peptostreptococcus_russellii_Calf135_1
Acetoanaerobium_1
Ruminococcus_flavefaciens_YRD2003_1
Ruminococcaceae_bacterium_P7_1
Clostridium_sp._DSM_8431_1
unclassified_Blautia_1
Blautia_sp._SF-50_1
Massilistercora_1
Lachnospiraceae_bacterium_G11_1
Streptococcus_gallolyticus_subsp._gallolyticus_1
Treponema_succinifaciens_1
Treponema_bryantii_XBD1002_1
Treponema_bryantii_NK4A124_1
0.00
0.01
0.02
0.03
0.04
0.05
factor(author)
duarte
hungate
lim2020
ours
refseq

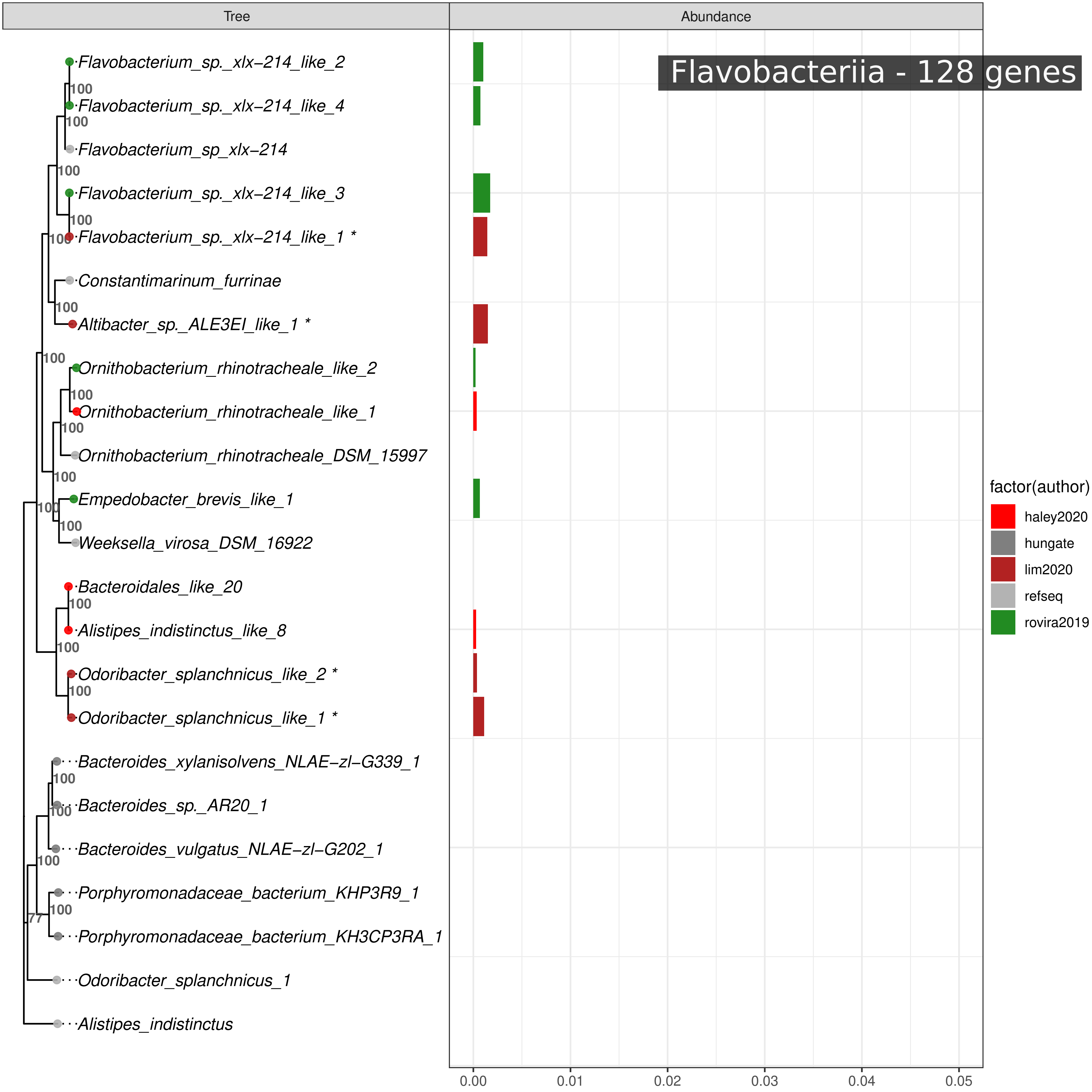
Tree
Abundance
Flavobacteriia - 128 genes
Flavobacterium_sp._xlx−214_like_2
Flavobacterium_sp._xlx−214_like_4
Flavobacterium_sp_xlx−214
Flavobacterium_sp._xlx−214_like_3
Flavobacterium_sp._xlx−214_like_1 *
Constantimarinum_furrinae
Altibacter_sp._ALE3EI_like_1 *
Ornithobacterium_rhinotracheale_like_2
Ornithobacterium_rhinotracheale_like_1
Ornithobacterium_rhinotracheale_DSM_15997
Empedobacter_brevis_like_1
Weeksella_virosa_DSM_16922
Bacteroidales_like_20
Alistipes_indistinctus_like_8
Odoribacter_splanchnicus_like_2 *
Odoribacter_splanchnicus_like_1 *
Bacteroides_xylanisolvens_NLAE−zl−G339_1
Bacteroides_sp._AR20_1
Bacteroides_vulgatus_NLAE−zl−G202_1
Porphyromonadaceae_bacterium_KHP3R9_1
Porphyromonadaceae_bacterium_KH3CP3RA_1
Odoribacter_splanchnicus_1
Alistipes_indistinctus
100
77
0.00
0.01
0.02
0.03
0.04
0.05
factor(author)
haley2020
hungate
lim2020
refseq
rovira2019

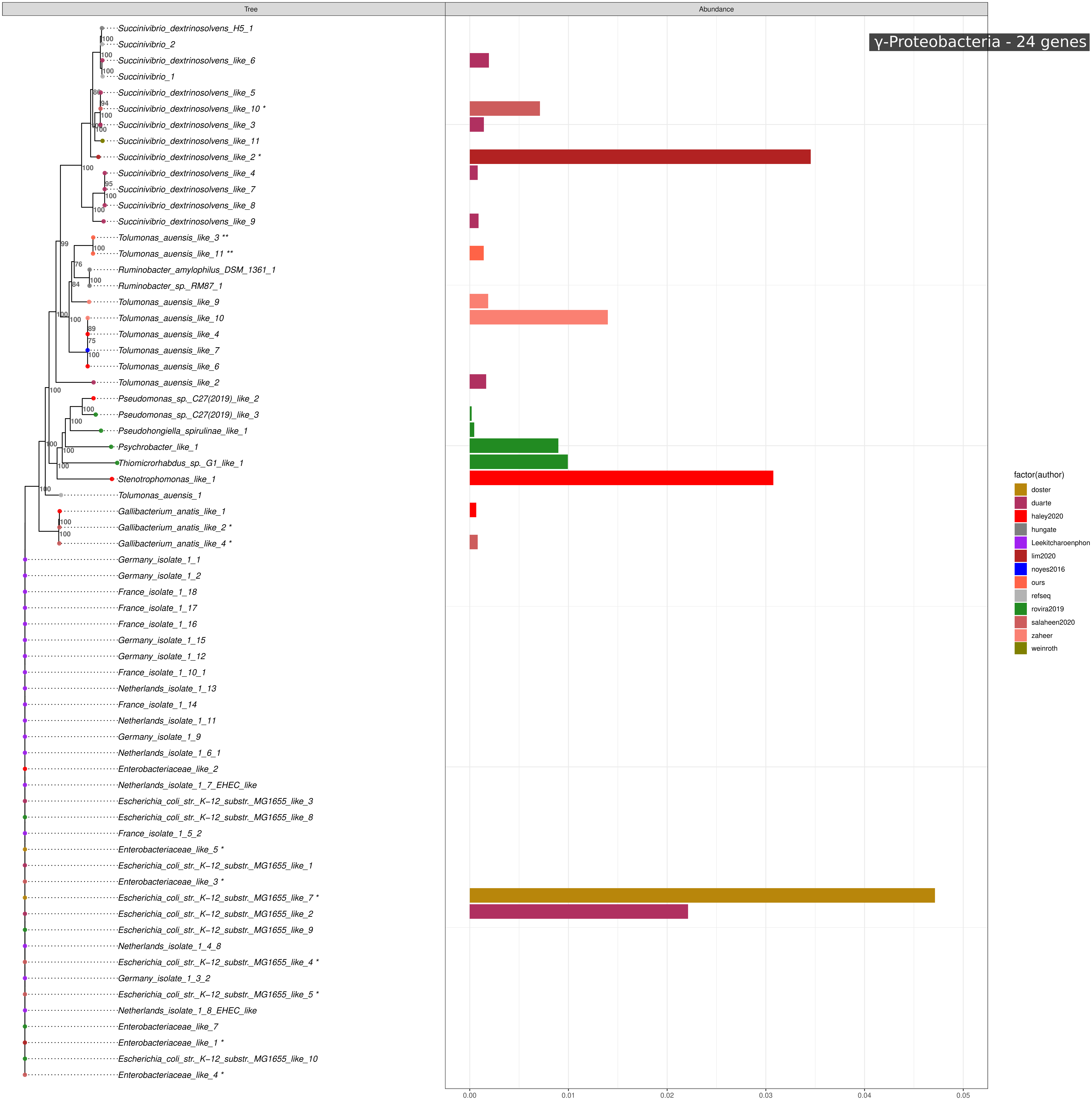
Tree
Abundance
γ-Proteobacteria - 24 genes
Succinivibrio_dextrinosolvens_H5_1
Succinivibrio_2
Succinivibrio_dextrinosolvens_like_6
Succinivibrio_1
Succinivibrio_dextrinosolvens_like_5
Succinivibrio_dextrinosolvens_like_10 *
Succinivibrio_dextrinosolvens_like_3
Succinivibrio_dextrinosolvens_like_11
Succinivibrio_dextrinosolvens_like_2 *
Succinivibrio_dextrinosolvens_like_4
Succinivibrio_dextrinosolvens_like_7
Succinivibrio_dextrinosolvens_like_8
Succinivibrio_dextrinosolvens_like_9
Tolumonas_auensis_like_3 **
Tolumonas_auensis_like_11 **
Ruminobacter_amylophilus_DSM_1361_1
Ruminobacter_sp._RM87_1
Tolumonas_auensis_like_9
Tolumonas_auensis_like_10
Tolumonas_auensis_like_4
Tolumonas_auensis_like_7
Tolumonas_auensis_like_6
Tolumonas_auensis_like_2
Pseudomonas_sp._C27(2019)_like_2
Pseudomonas_sp._C27(2019)_like_3
Pseudohongiella_spirulinae_like_1
Psychrobacter_like_1
Thiomicrorhabdus_sp._G1_like_1
Stenotrophomonas_like_1
Tolumonas_auensis_1
Gallibacterium_anatis_like_1
Gallibacterium_anatis_like_2 *
Gallibacterium_anatis_like_4 *
Germany_isolate_1_1
Germany_isolate_1_2
France_isolate_1_18
France_isolate_1_17
France_isolate_1_16
Germany_isolate_1_15
Germany_isolate_1_12
France_isolate_1_10_1
Netherlands_isolate_1_13
France_isolate_1_14
Netherlands_isolate_1_11
Germany_isolate_1_9
Netherlands_isolate_1_6_1
Enterobacteriaceae_like_2
Netherlands_isolate_1_7_EHEC_like
Escherichia_coli_str._K−12_substr._MG1655_like_3
Escherichia_coli_str._K−12_substr._MG1655_like_8
France_isolate_1_5_2
Enterobacteriaceae_like_5 *
Escherichia_coli_str._K−12_substr._MG1655_like_1
Enterobacteriaceae_like_3 *
Escherichia_coli_str._K−12_substr._MG1655_like_7 *
Escherichia_coli_str._K−12_substr._MG1655_like_2
Escherichia_coli_str._K−12_substr._MG1655_like_9
Netherlands_isolate_1_4_8
Escherichia_coli_str._K−12_substr._MG1655_like_4 *
Germany_isolate_1_3_2
Escherichia_coli_str._K−12_substr._MG1655_like_5 *
Netherlands_isolate_1_8_EHEC_like
Enterobacteriaceae_like_7
Enterobacteriaceae_like_1 *
Escherichia_coli_str._K−12_substr._MG1655_like_10
Enterobacteriaceae_like_4 *
0.00
0.01
0.02
0.03
0.04
0.05
factor(author)
doster
duarte
haley2020
hungate
Leekitcharoenphon
lim2020
noyes2016
ours
refseq
rovira2019
salaheen2020
zaheer
weinroth

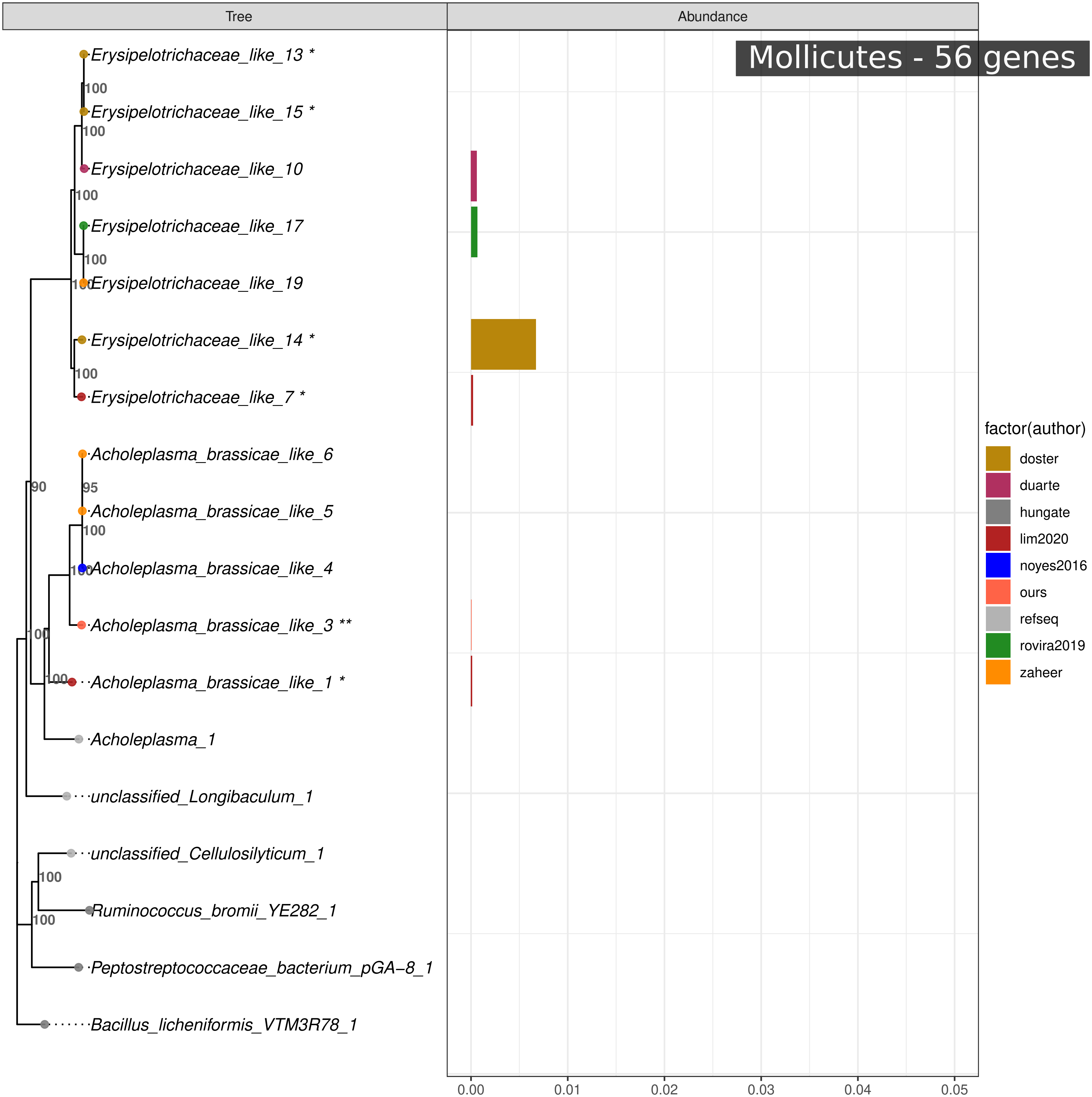

Tree
Abundance
Mollicutes - 56 genes
Erysipelotrichaceae_like_13 *
Erysipelotrichaceae_like_15 *
Erysipelotrichaceae_like_10
Erysipelotrichaceae_like_17
Erysipelotrichaceae_like_19
Erysipelotrichaceae_like_14 *
Erysipelotrichaceae_like_7 *
Acholeplasma_brassicae_like_6
Acholeplasma_brassicae_like_5
Acholeplasma_brassicae_like_4
Acholeplasma_brassicae_like_3 **
Acholeplasma_brassicae_like_1 *
Acholeplasma_1
unclassified_Longibaculum_1
unclassified_Cellulosilyticum_1
Ruminococcus_bromii_YE282_1
Peptostreptococcaceae_bacterium_pGA-8_1
Bacillus_licheniformis_VTM3R78_1
100
100
100
100
100
90
95
100
100
100
100
100
100
0.00
0.01
0.02
0.03
0.04
0.05
factor(author)
doster
duarte
hungate
lim2020
noyes2016
ours
refseq
rovira2019
zaheer

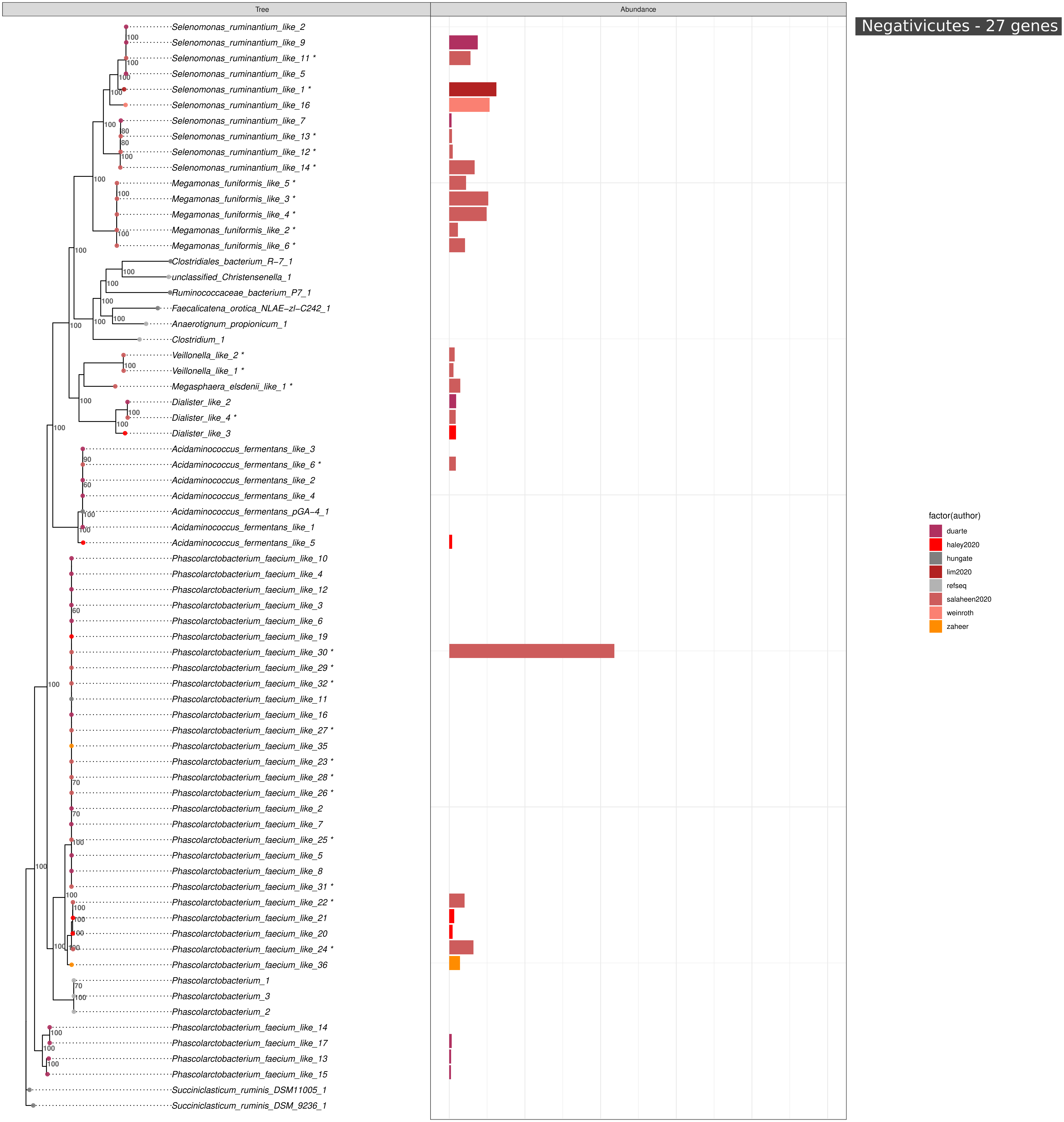

Tree
Abundance
Negativicutes - 27 genes
Selenomonas_ruminantium_like_2
Selenomonas_ruminantium_like_9
Selenomonas_ruminantium_like_11 *
Selenomonas_ruminantium_like_5
Selenomonas_ruminantium_like_1 *
Selenomonas_ruminantium_like_16
Selenomonas_ruminantium_like_7
Selenomonas_ruminantium_like_13 *
Selenomonas_ruminantium_like_12 *
Selenomonas_ruminantium_like_14 *
Megamonas_funiformis_like_5 *
Megamonas_funiformis_like_3 *
Megamonas_funiformis_like_4 *
Megamonas_funiformis_like_2 *
Megamonas_funiformis_like_6 *
Clostridiales_bacterium_R-7_1
unclassified_Christensenella_1
Ruminococcaceae_bacterium_P7_1
Faecalicatena_orotica_NLAE-zl-C242_1
Anaerotignum_propionicum_1
Clostridium_1
Veillonella_like_2 *
Veillonella_like_1 *
Megasphaera_elsdenii_like_1 *
Dialister_like_2
Dialister_like_4 *
Dialister_like_3
Acidaminococcus_fermentans_like_3
Acidaminococcus_fermentans_like_6 *
Acidaminococcus_fermentans_like_2
Acidaminococcus_fermentans_like_4
Acidaminococcus_fermentans_pGA-4_1
Acidaminococcus_fermentans_like_1
Acidaminococcus_fermentans_like_5
Phascolarctobacterium_faecium_like_10
Phascolarctobacterium_faecium_like_4
Phascolarctobacterium_faecium_like_12
Phascolarctobacterium_faecium_like_3
Phascolarctobacterium_faecium_like_6
Phascolarctobacterium_faecium_like_19
Phascolarctobacterium_faecium_like_30 *
Phascolarctobacterium_faecium_like_29 *
Phascolarctobacterium_faecium_like_32 *
Phascolarctobacterium_faecium_like_11
Phascolarctobacterium_faecium_like_16
Phascolarctobacterium_faecium_like_27 *
Phascolarctobacterium_faecium_like_35
Phascolarctobacterium_faecium_like_23 *
Phascolarctobacterium_faecium_like_28 *
Phascolarctobacterium_faecium_like_26 *
Phascolarctobacterium_faecium_like_2
Phascolarctobacterium_faecium_like_7
Phascolarctobacterium_faecium_like_25 *
Phascolarctobacterium_faecium_like_5
Phascolarctobacterium_faecium_like_8
Phascolarctobacterium_faecium_like_31 *
Phascolarctobacterium_faecium_like_22 *
Phascolarctobacterium_faecium_like_21
Phascolarctobacterium_faecium_like_20
Phascolarctobacterium_faecium_like_24 *
Phascolarctobacterium_faecium_like_36
Phascolarctobacterium_1
Phascolarctobacterium_3
Phascolarctobacterium_2
Phascolarctobacterium_faecium_like_14
Phascolarctobacterium_faecium_like_17
Phascolarctobacterium_faecium_like_13
Phascolarctobacterium_faecium_like_15
Succiniclasticum_ruminis_DSM11005_1
Succiniclasticum_ruminis_DSM_9236_1
factor(author)
duarte
haley2020
hungate
lim2020
refseq
salaheen2020
weinroth
zaheer

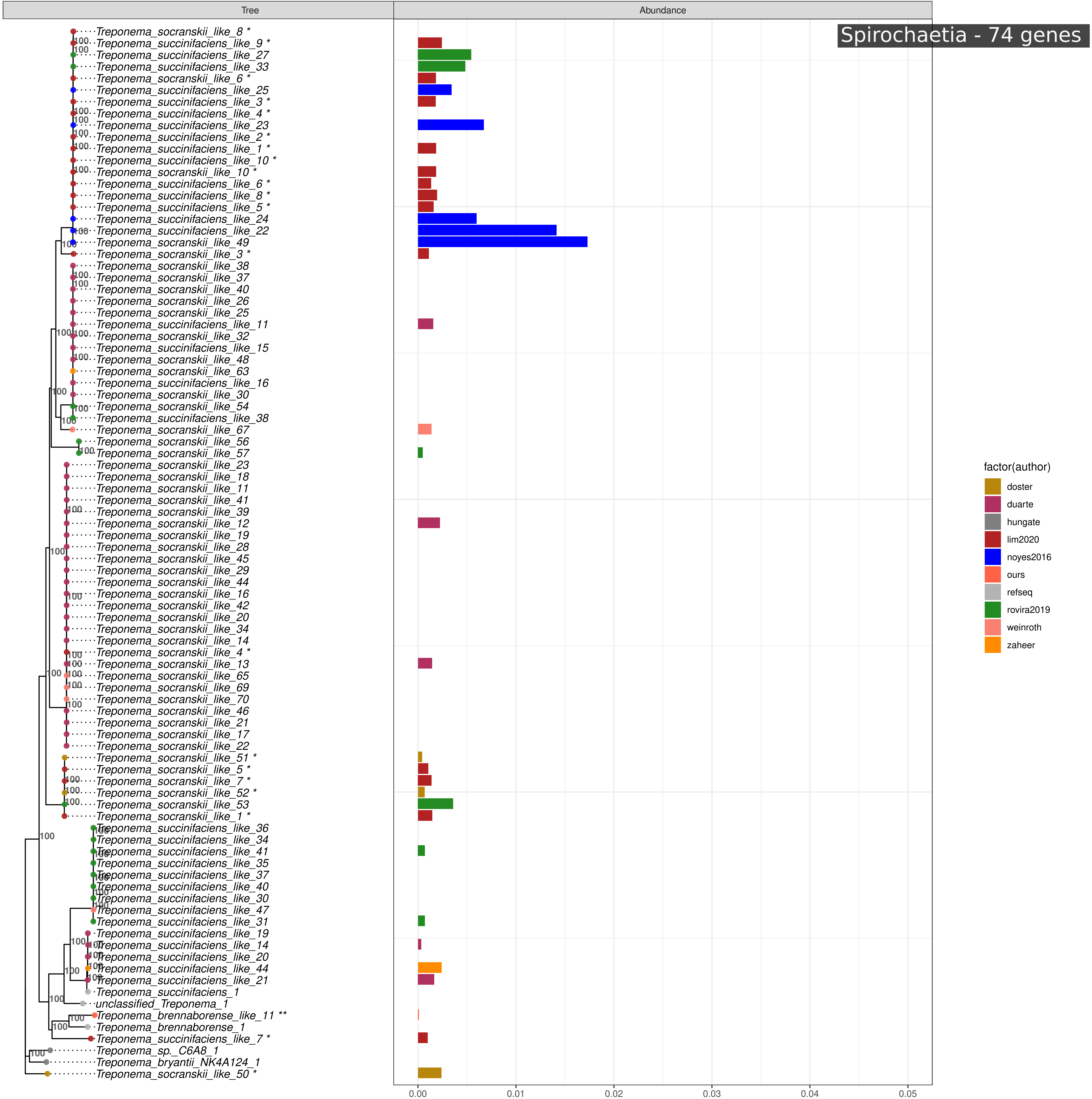
Tree
Abundance
Spirochaetia - 74 genes
Treponema_socranskii_like_8 *
Treponema_succinifaciens_like_9 *
Treponema_succinifaciens_like_27
Treponema_succinifaciens_like_33
Treponema_socranskii_like_6 *
Treponema_succinifaciens_like_25
Treponema_succinifaciens_like_3 *
Treponema_succinifaciens_like_4 *
Treponema_succinifaciens_like_23
Treponema_succinifaciens_like_2 *
Treponema_succinifaciens_like_1 *
Treponema_succinifaciens_like_10 *
Treponema_socranskii_like_10 *
Treponema_succinifaciens_like_6 *
Treponema_succinifaciens_like_8 *
Treponema_succinifaciens_like_5 *
Treponema_succinifaciens_like_24
Treponema_succinifaciens_like_22
Treponema_socranskii_like_49
Treponema_socranskii_like_3 *
Treponema_socranskii_like_38
Treponema_socranskii_like_37
Treponema_socranskii_like_40
Treponema_socranskii_like_26
Treponema_socranskii_like_25
Treponema_succinifaciens_like_11
Treponema_socranskii_like_32
Treponema_succinifaciens_like_15
Treponema_socranskii_like_48
Treponema_socranskii_like_63
Treponema_succinifaciens_like_16
Treponema_socranskii_like_30
Treponema_socranskii_like_54
Treponema_succinifaciens_like_38
Treponema_socranskii_like_67
Treponema_socranskii_like_56
Treponema_socranskii_like_57
Treponema_socranskii_like_23
Treponema_socranskii_like_18
Treponema_socranskii_like_11
Treponema_socranskii_like_41
Treponema_socranskii_like_39
Treponema_socranskii_like_12
Treponema_socranskii_like_19
Treponema_socranskii_like_28
Treponema_socranskii_like_45
Treponema_socranskii_like_29
Treponema_socranskii_like_44
Treponema_socranskii_like_16
Treponema_socranskii_like_42
Treponema_socranskii_like_20
Treponema_socranskii_like_34
Treponema_socranskii_like_14
Treponema_socranskii_like_4 *
Treponema_socranskii_like_13
Treponema_socranskii_like_65
Treponema_socranskii_like_69
Treponema_socranskii_like_70
Treponema_socranskii_like_46
Treponema_socranskii_like_21
Treponema_socranskii_like_17
Treponema_socranskii_like_22
Treponema_socranskii_like_51 *
Treponema_socranskii_like_5 *
Treponema_socranskii_like_7 *
Treponema_socranskii_like_52 *
Treponema_socranskii_like_53
Treponema_socranskii_like_1 *
Treponema_succinifaciens_like_36
Treponema_succinifaciens_like_34
Treponema_succinifaciens_like_41
Treponema_succinifaciens_like_35
Treponema_succinifaciens_like_37
Treponema_succinifaciens_like_40
Treponema_succinifaciens_like_30
Treponema_succinifaciens_like_47
Treponema_succinifaciens_like_31
Treponema_succinifaciens_like_19
Treponema_succinifaciens_like_14
Treponema_succinifaciens_like_20
Treponema_succinifaciens_like_44
Treponema_succinifaciens_like_21
Treponema_succinifaciens_1
unclassified_Treponema_1
Treponema_brennaborense_like_11 **
Treponema_brennaborense_1
Treponema_succinifaciens_like_7 *
Treponema_sp._C6A8_1
Treponema_bryantii_NK4A124_1
Treponema_socranskii_like_50 *
0.00
0.01
0.02
0.03
0.04
0.05
factor(author)
doster
duarte
hungate
lim2020
noyes2016
ours
refseq
rovira2019
weinroth
zaheer

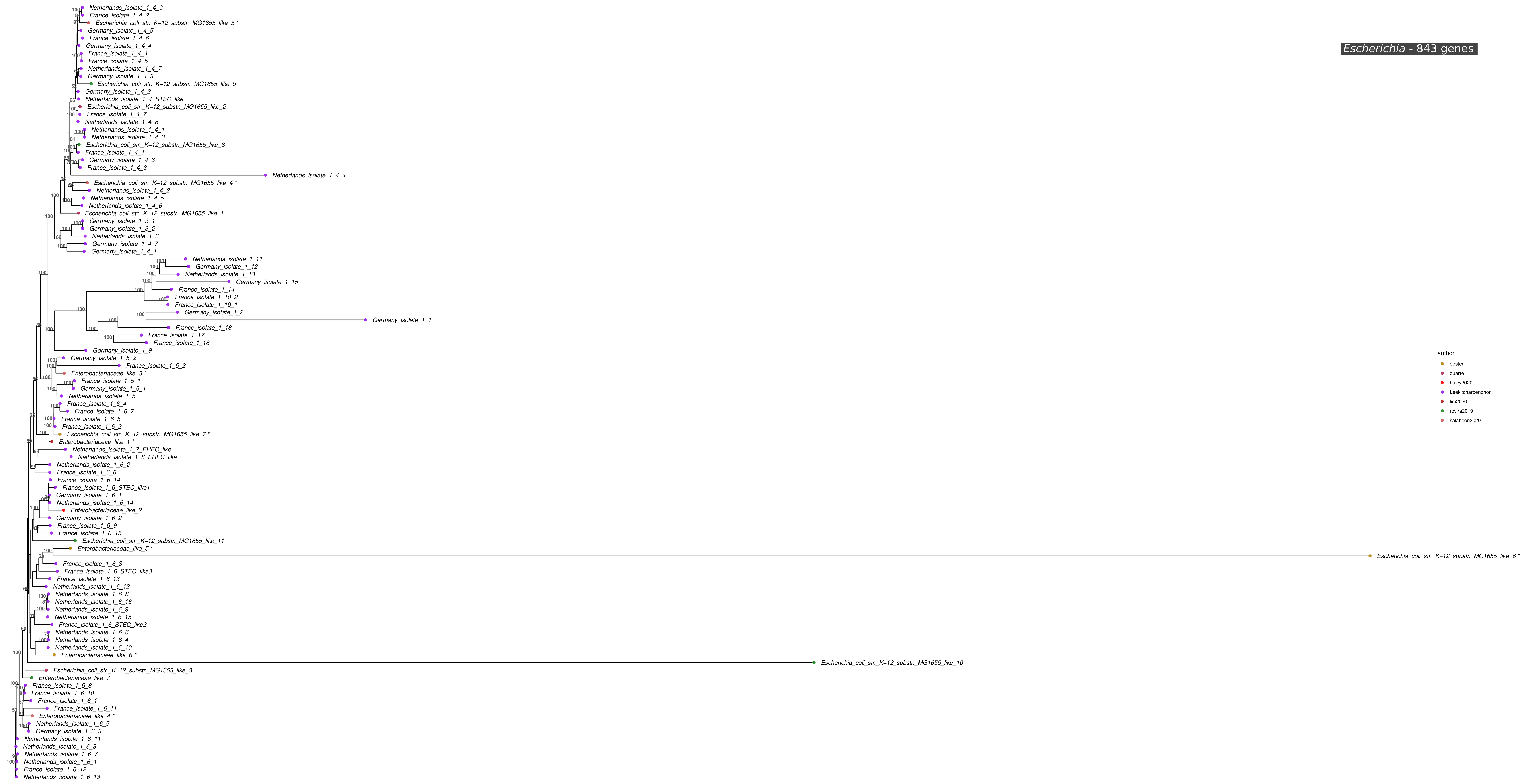
Escherichia - 843 genes
Netherlands_isolate_1_4_9
France_isolate_1_4_2
Escherichia_coli_str._K-12_substr._MG1655_like_5 *
Germany_isolate_1_4_5
France_isolate_1_4_6
Germany_isolate_1_4_4
France_isolate_1_4_4
France_isolate_1_4_5
Netherlands_isolate_1_4_7
Germany_isolate_1_4_3
Escherichia_coli_str._K-12_substr._MG1655_like_9
Germany_isolate_1_4_2
Netherlands_isolate_1_4_STEC_like
Escherichia_coli_str._K-12_substr._MG1655_like_2
France_isolate_1_4_7
Netherlands_isolate_1_4_8
Netherlands_isolate_1_4_1
Netherlands_isolate_1_4_3
Escherichia_coli_str._K-12_substr._MG1655_like_8
France_isolate_1_4_1
Germany_isolate_1_4_6
France_isolate_1_4_3
Netherlands_isolate_1_4_4
Escherichia_coli_str._K-12_substr._MG1655_like_4 *
Netherlands_isolate_1_4_2
Netherlands_isolate_1_4_5
Netherlands_isolate_1_4_6
Escherichia_coli_str._K-12_substr._MG1655_like_1
Germany_isolate_1_3_1
Germany_isolate_1_3_2
Netherlands_isolate_1_3
Germany_isolate_1_4_7
Germany_isolate_1_4_1
Netherlands_isolate_1_11
Germany_isolate_1_12
Netherlands_isolate_1_13
Germany_isolate_1_15
France_isolate_1_14
France_isolate_1_10_2
France_isolate_1_10_1
Germany_isolate_1_2
Germany_isolate_1_1
France_isolate_1_18
France_isolate_1_17
France_isolate_1_16
Germany_isolate_1_9
Germany_isolate_1_5_2
France_isolate_1_5_2
Enterobacteriaceae_like_3 *
France_isolate_1_5_1
Germany_isolate_1_5_1
Netherlands_isolate_1_5
France_isolate_1_6_4
France_isolate_1_6_7
France_isolate_1_6_5
France_isolate_1_6_2
Escherichia_coli_str._K-12_substr._MG1655_like_7 *
Enterobacteriaceae_like_1 *
Netherlands_isolate_1_7_EHEC_like
Netherlands_isolate_1_8_EHEC_like
Netherlands_isolate_1_6_2
France_isolate_1_6_6
France_isolate_1_6_14
France_isolate_1_6_STEC_like1
Germany_isolate_1_6_1
Netherlands_isolate_1_6_14
Enterobacteriaceae_like_2
Germany_isolate_1_6_2
France_isolate_1_6_9
France_isolate_1_6_15
Escherichia_coli_str._K-12_substr._MG1655_like_11
Enterobacteriaceae_like_5 *
Escherichia_coli_str._K-12_substr._MG1655_like_6 *
France_isolate_1_6_3
France_isolate_1_6_STEC_like3
France_isolate_1_6_13
Netherlands_isolate_1_6_12
Netherlands_isolate_1_6_8
Netherlands_isolate_1_6_16
Netherlands_isolate_1_6_9
Netherlands_isolate_1_6_15
France_isolate_1_6_STEC_like2
Netherlands_isolate_1_6_6
Netherlands_isolate_1_6_4
Netherlands_isolate_1_6_10
Enterobacteriaceae_like_6 *
Escherichia_coli_str._K-12_substr._MG1655_like_10
Escherichia_coli_str._K-12_substr._MG1655_like_3
Enterobacteriaceae_like_7
France_isolate_1_6_8
France_isolate_1_6_10
France_isolate_1_6_1
France_isolate_1_6_11
Enterobacteriaceae_like_4 *
Netherlands_isolate_1_6_5
Germany_isolate_1_6_3
Netherlands_isolate_1_6_11
Netherlands_isolate_1_6_3
Netherlands_isolate_1_6_7
Netherlands_isolate_1_6_1
France_isolate_1_6_12
Netherlands_isolate_1_6_13
author
doster
duarte
haley2020
Leekitcharoenphon
lim2020
rovira2019
salaheen2020

Common tree - 13 genes
MAGs >99% completeness, contamination <5%

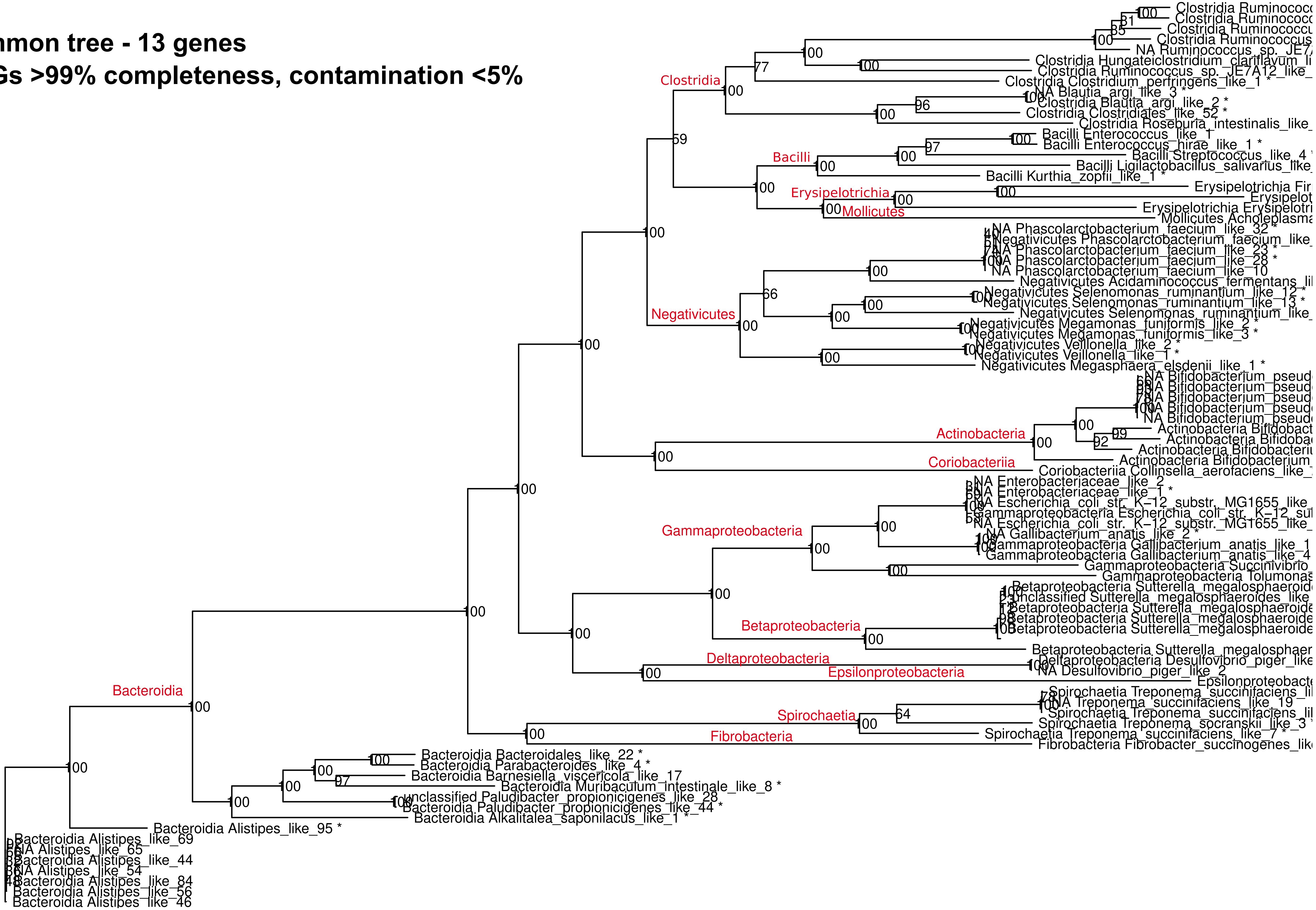

Supplement: Supplementary file 12 — Additional file 11: Supplementary Results 1. Phylogenies built in our study on the basis of 13-843 AA sequences of single copy orthologs. The first fifteen panels present the class specific phylogenies for the 2114 MAGs identified in our study and their close relatives. Bootstrap support is presented (light gray next to branches) and colored circles show study ID of the sample that each MAG was generated from. The light gray and dark gray circles show genomes from the reference databases (Hungate 1000 and RefSeq respectively). At the upper right corner of each phylogeny, the class and number of genes used for concatenated alignments are presented. Single asterisks next to names mark MAGs that originate from fecal samples that were collected from single animals and double asterisks MAGs that originate from fecal samples collected for this study. Average abundance for each MAG was calculated by first summing the abundance of all contigs of a MAG per sample (calculated by KMA) and then by estimating the mean across all samples. The last two panels present the Escherichia specific phylogeny built using MAGs built in our study and Escherichia isolates sequenced in a previous study [23] and a global phylogeny (labeled as «common tree») built using 87 high quality MAGs (>99% completeness), which are representatives of most bacterial classes. Signs with exclamation marks in the phylogeny of Escherichia mark isolates carrying virulence genes. Bacterial classes are displayed with a red font in the ‘common tree’ phylogeny. [file 40168_2022_1357_MOESM11_ESM.pdf]
