# Supplementary material for: A global phylogenomic and metabolic reconstruction of the large intestine bacterial community of domesticated cattle
Source: Microbiome. 2022 Sep 26;10:155. doi: 10.1186/s40168-022-01357-1 (PMC9511753; doi:10.1186/s40168-022-01357-1)
Supplement: Supplementary file 13 — Additional file 12: Supplementary Results 2. Summary of the most common KEGG metabolic pathways illustrations (which are complete in at least one MAG). Pathways are presented for the common (80% of 210 individual sample and most abundant; Table S6) contigs (A), the contrast between winter and summer samples in our study (B) and the contrast between animals before and after introduction of a solid feed (C) (Table S8). The illustrations present the parts of the metabolic pathways that show specialization (i.e. import of compounds) and not common/housekeeping parts (e.g. catabolism of glucose to pyruvate or the Entner–Doudoroff pathway) for simplification. In addition, parts of the pathways that are likely linked to housekeeping (e.g. cell wall biosynthesis), pathogenesis and other non-metabolic functions have been omitted. [file 40168_2022_1357_MOESM12_ESM.pdf]

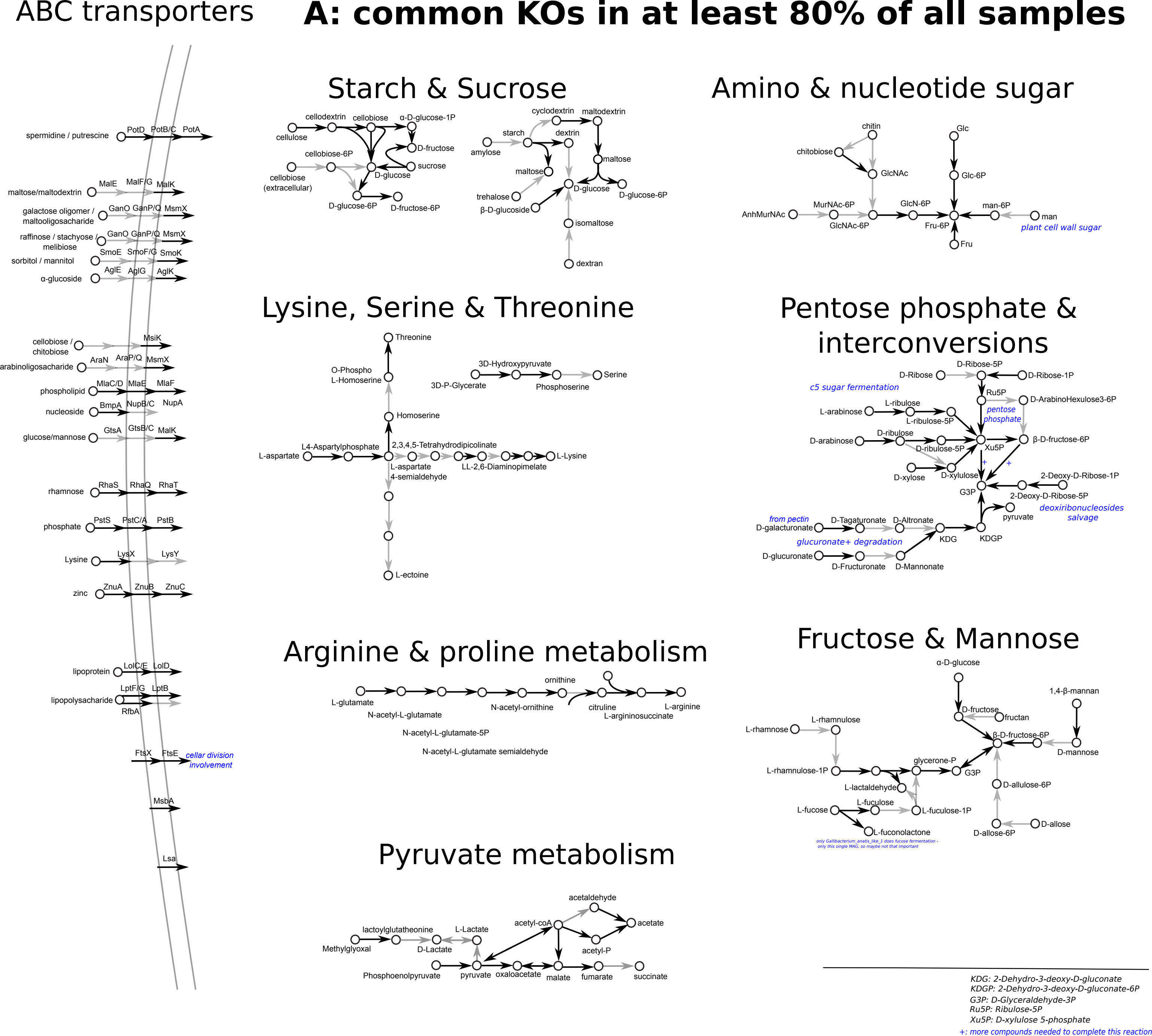
A: common KOs in at least 80% of all samples
ABC transporters
spermidine / putrescine
PotD
PotB/C
PotA
maltose/maltodextrin
MalE
MalF/G
MalK
galactose oligomer / maltooligosacharide
GanO
GanP/Q
MsmX
raffinose / stachyose / melibiose
GanO
GanP/Q
MsmX
sorbitol / mannitol
SmoE
SmoF/G
SmoK
α-glucoside
AglE
AglG
AglK
cellobiose / chitobiose
MsiK
arabinoligosacharide
AraN
AraP/Q
MsmX
phospholipid
MlaC/D
MlaE
MlaF
nucleoside
BmpA
NupB/C
NupA
glucose/mannose
GtsA
GtsB/C
MalK
rhamnose
RhaS
RhaQ
RhaT
phosphate
PstS
PstC/A
PstB
Lysine
LysX
LysY
zinc
ZnuA
ZnuB
ZnuC
lipoprotein
LolC/E
LolD
lipopolysacharide
LptF/G
LptB
RfbA
FtsX
FtsE
cellar division involvement
MsbA
Lsa
Starch & Sucrose
cellodextrin
cellobiose
α-D-glucose-1P
cellulose
D-fructose
cellobiose-6P
sucrose
cellobiose (extracellular)
D-glucose
D-glucose-6P
D-fructose-6P
cyclodextrin
maltodextrin
starch
dextrin
amylose
maltose
maltose
trehalose
D-glucose
D-glucose-6P
β-D-glucoside
isomaltose
dextran
Amino & nucleotide sugar
chitin
Glc
chitobiose
GlcNAc
Glc-6P
MurNAc-6P
GlcN-6P
man-6P
AnhMurNAc
man
GlcNAc-6P
Fru-6P
plant cell wall sugar
Fru
Lysine, Serine & Threonine
Threonine
O-Phospho L-Homoserine
3D-Hydroxypyruvate
3D-P-Glycerate
Serine
Phosphoserine
Homoserine
L4-Aspartylphosphate
2,3,4,5-Tetrahydrodipicolinate
L-aspartate
L-Lysine
L-aspartate 4-semialdehyde
LL-2,6-Diaminopimelate
L-ectoine
Pentose phosphate & interconversions
D-Ribose-5P
D-Ribose
D-Ribose-1P
c5 sugar fermentation
Ru5P
D-ArabinoHexulose3-6P
L-arabinose
L-ribulose
L-ribulose-5P
pentose phosphate
D-ribulose
D-arabinose
D-ribulose-5P
β-D-fructose-6P
Xu5P
D-xylose
D-xylulose
+
+
2-Deoxy-D-Ribose-1P
G3P
2-Deoxy-D-Ribose-5P
pyruvate
deoxiribonucleosides salvage
from pectin
D-galacturonate
D-Tagaturonate
D-Altronate
KDG
KDGP
glucuronate+ degradation
D-glucuronate
D-Fructuronate
D-Mannonate
Arginine & proline metabolism
ornithine
L-glutamate
N-acetyl-L-glutamate
N-acetyl-ornithine
citruline
L-argininosuccinate
L-arginine
N-acetyl-L-glutamate-5P
N-acetyl-L-glutamate semialdehyde
Fructose & Mannose
α-D-glucose
1,4-β-mannan
D-fructose
fructan
L-rhamnulose
L-rhamnose
β-D-fructose-6P
D-mannose
glycerone-P
L-rhamnulose-1P
G3P
L-lactaldehyde
D-allulose-6P
L-fucose
L-fuculose
L-fuculose-1P
L-fuconolactone
D-allose-6P
D-allose
only Gallibacterium_anatis_like_1 does fucose fermentation - only this single MAG, so maybe not that important
Pyruvate metabolism
acetaldehyde
acetyl-coA
acetate
lactoylglutatheonine
L-Lactate
Methylglyoxal
D-Lactate
acetyl-P
Phosphoenolpyruvate
pyruvate
oxaloacetate
malate
fumarate
succinate
KDG: 2-Dehydro-3-deoxy-D-gluconate
KDGP: 2-Dehydro-3-deoxy-D-gluconate-6P
G3P: D-Glyceraldehyde-3P
Ru5P: Ribulose-5P
Xu5P: D-xylulose 5-phosphate
+: more compounds needed to complete this reaction

B: Diff. rep KOs in summer and winter samples from Theix

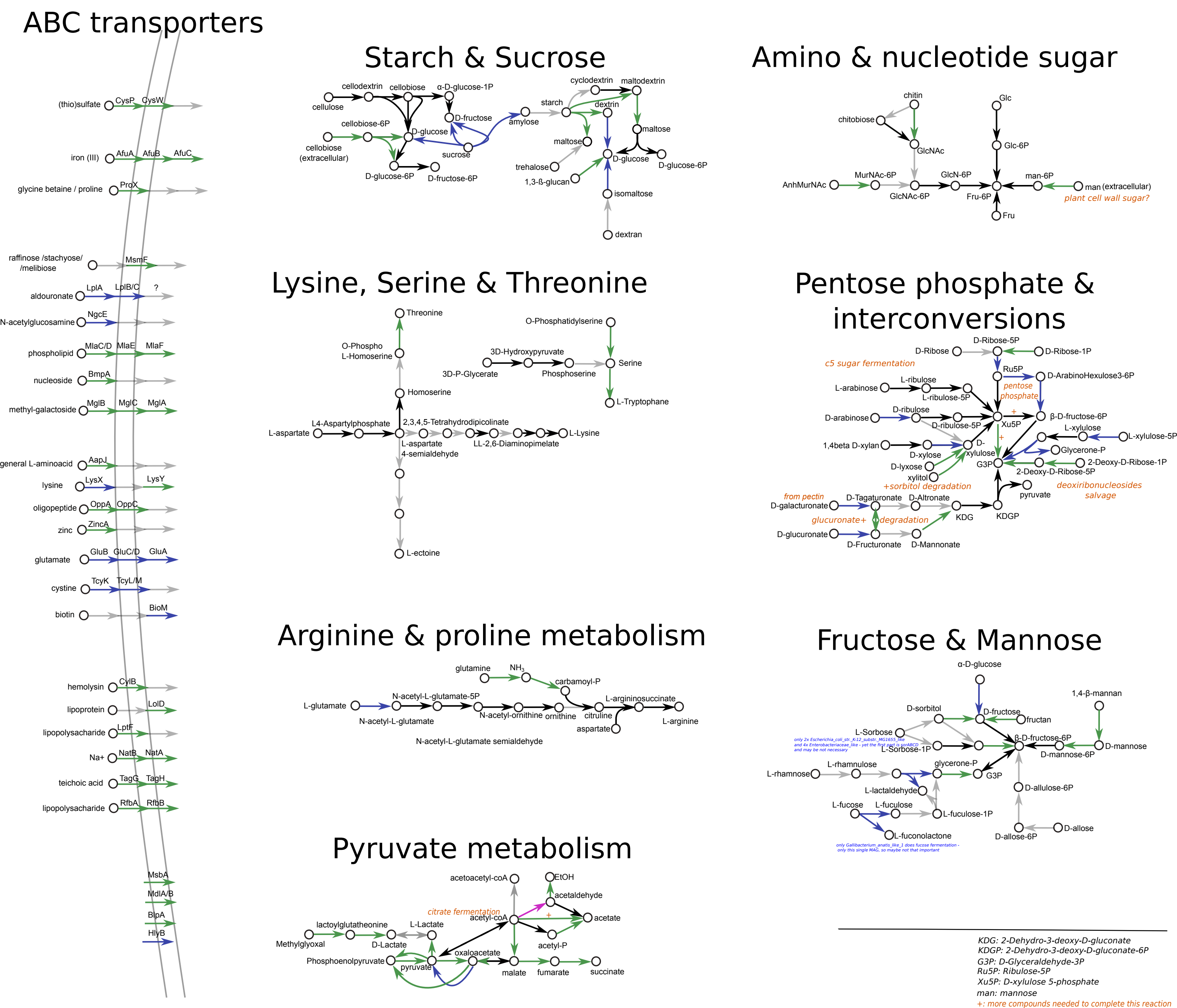

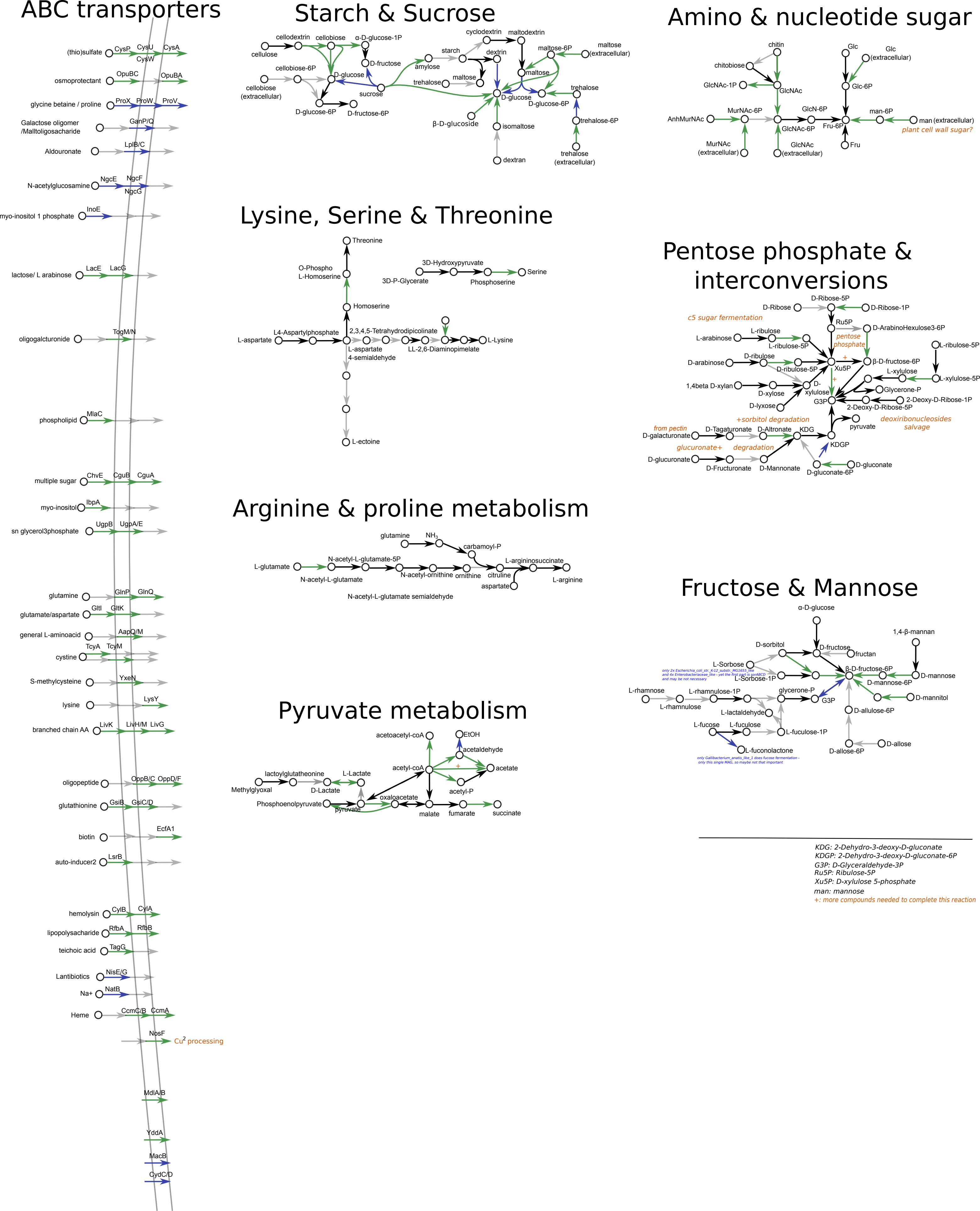

ABC transporters
(thio)sulfate
CysP
CysU
CysW
CysA
osmoprotectant
OpuBC
OpuBA
glycine betaine / proline
ProX
ProW
ProV
Galactose oligomer
/Malltoligosacharide
GanP/Q
Aldouronate
LplB/C
N-acetylglucosamine
NgcE
NgcF
NgcG
myo-inositol 1 phosphate
InoE
lactose/ L arabinose
LacE
LacG
oligogalcturonide
TogM/N
phospholipid
MlaC
multiple sugar
ChvE
CguB
CguA
myo-inositol
IbpA
sn glycerol3phosphate
UgpB
UgpA/E
glutamine
GlnP
GlnQ
glutamate/aspartate
GltI
GltK
general L-aminoacid
AapQ/M
cystine
TcyA
TcyM
S-methylcysteine
YxeN
lysine
LysY
branched chain AA
LivK
LivH/M
LivG
oligopeptide
OppB/C
OppD/F
glutathionine
GsiB
GsiC/D
biotin
EcfA1
auto-inducer2
LsrB
hemolysin
CylB
CylA
lipopolysacharide
RfbA
RfbB
teichoic acid
TagG
Lantibiotics
NisE/G
Na+
NatB
Heme
CcmC/B
CcmA
NosF
Cu^2 processing
MdlA/B
YddA
MacB
CydC/D
Starch & Sucrose
cellodextrin
cellobiose
α-D-glucose-1P
cellulose
D-fructose
cellobiose-6P
D-glucose
cellobiose
(extracellular)
sucrose
D-glucose-6P
D-fructose-6P
cyclodextrin
maltodextrin
starch
dextrin
amylose
maltose-6P
maltose
(extracellular)
maltose
trehalose
maltose
D-glucose
D-glucose-6P
trehalose
β-D-glucoside
isomaltose
trehalose-6P
dextran
trehalose
(extracellular)
Amino & nucleotide sugar
chitin
chitobiose
GlcNAc-1P
GlcNAc
AnhMurNAc
MurNAc-6P
GlcN-6P
GlcNAc-6P
MurNAc
(extracellular)
GlcNAc
(extracellular)
Glc
Glc
(extracellular)
Glc-6P
Fru-6P
man-6P
man (extracellular)
Fru
plant cell wall sugar?
Lysine, Serine & Threonine
Threonine
O-Phospho
L-Homoserine
Homoserine
3D-Hydroxypyruvate
3D-P-Glycerate
Phosphoserine
Serine
L4-Aspartylphosphate
2,3,4,5-Tetrahydrodipicolinate
L-aspartate
L-aspartate
4-semialdehyde
LL-2,6-Diaminopimelate
L-Lysine
L-ectoine
Pentose phosphate &
interconversions
D-Ribose-5P
D-Ribose
D-Ribose-1P
c5 sugar fermentation
Ru5P
D-ArabinoHexulose3-6P
L-ribulose
L-arabinose
L-ribulose-5P
pentose
phosphate
L-ribulose-5P
D-ribulose
D-arabinose
D-ribulose-5P
Xu5P
β-D-fructose-6P
L-xylulose
L-xylulose-5P
1,4beta D-xylan
D-xylose
D-
xylulose
Glycerone-P
D-lyxose
G3P
2-Deoxy-D-Ribose-1P
2-Deoxy-D-Ribose-5P
+sorbitol degradation
pyruvate
deoxiribonucleosides
salvage
from pectin
D-galacturonate
D-Tagaturonate
D-Altronate
KDG
glucuronate+
degradation
KDGP
D-glucuronate
D-Fructuronate
D-Mannonate
D-gluconate-6P
D-gluconate
Arginine & proline metabolism
glutamine
NH_3
carbamoyl-P
L-glutamate
N-acetyl-L-glutamate-5P
L-argininosuccinate
N-acetyl-L-glutamate
N-acetyl-ornithine
ornithine
citruline
L-arginine
aspartate
N-acetyl-L-glutamate semialdehyde
Fructose & Mannose
α-D-glucose
1,4-β-mannan
D-sorbitol
D-fructose
fructan
L-Sorbose
β-D-fructose-6P
L-Sorbose-1P
D-mannose
D-mannose-6P
L-rhamnose
L-rhamnulose-1P
glycerone-P
L-rhamnulose
G3P
D-mannitol
L-lactaldehyde
D-allulose-6P
L-fucose
L-fuculose
L-fuculose-1P
L-fuconolactone
D-allose
D-allose-6P
Pyruvate metabolism
acetoacetyl-coA
EtOH
acetaldehyde
acetyl-coA
acetate
lactoylglutatheonine
L-Lactate
Methylglyoxal
D-Lactate
acetyl-P
Phosphoenolpyruvate
oxaloacetate
pyruvate
malate
fumarate
succinate
KDG: 2-Dehydro-3-deoxy-D-gluconate
KDGP: 2-Dehydro-3-deoxy-D-gluconate-6P
G3P: D-Glyceraldehyde-3P
Ru5P: Ribulose-5P
Xu5P: D-xylulose 5-phosphate
man: mannose
+: more compounds needed to complete this reaction
