# Supplementary material for: A global phylogenomic and metabolic reconstruction of the large intestine bacterial community of domesticated cattle
Source: Microbiome. 2022 Sep 26;10:155. doi: 10.1186/s40168-022-01357-1 (PMC9511753; doi:10.1186/s40168-022-01357-1)
Supplement: Supplementary file 14 — Additional file 13: Supplementary Results 3. There are two main approaches to identify zoonotic and animal pathogens in fecal (or other) samples: a culture-based approach that typically isolates microbial strains and then examines their virulence potential using PCR or sequencing (individual genome sequencing) and a shotgun metagenomic approach that examines all sequencing reads against a set of known virulence genes (e.g. VFdb database). A common controversy between the two methods is that the metagenomic approach likely overestimates the diversity of community members and the diversity of virulence or other microbial genes, while the culture-based approach underestimates it. However, many microbes cannot be cultivated simply because their niches are too specific. As a result of that, in any given sample there is always a number of impossible-to-cultivate microbes that will not be detected. On the other hand, shotgun metagenomic approaches are also prone to errors because they identify any sequences that are present in the samples regardless of whether they come from microbes that are present in the sample or sequences from dead microbes. Despite how the tendency to miscalculate gene diversity of the two approaches is in opposite directions, it has always been assumed that their results (when examining the same samples) should largely be in agreement. Fifteen of the samples that we used, originating from composite samples of feces, had been used for shotgun metagenomics sequencing [26] and for isolating Escherichia strains and independent genome sequencing [23]. Therefore, we took advantage of that unique opportunity to test the above hypothesis. Using the metagenomic reads from the fifteen samples as well as the genome sequences from the 150 Escherichia isolates (for each fecal sample ten Escherichia isolates were genome sequenced; [23]), we mapped the predicted ORFs from both datasets to the entire VFdb database (not the pathogenic subset we used in the manuscrip [file 40168_2022_1357_MOESM13_ESM.pdf]

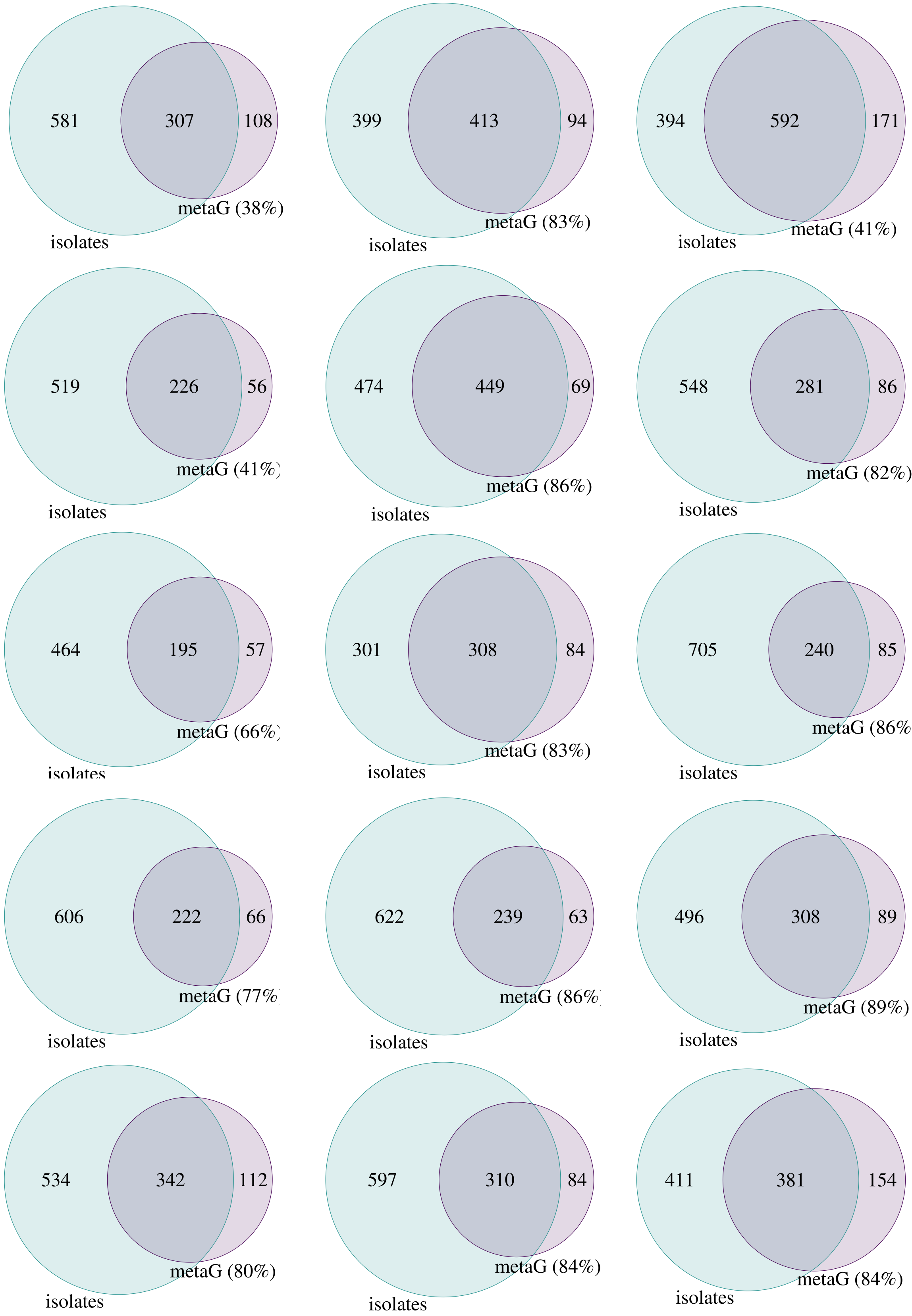

581
307
108
metaG (38%)
isolates
399
413
94
metaG (83%)
isolates
394
592
171
metaG (41%)
isolates
519
226
56
metaG (41%
isolates
474
449
69
metaG (86%)
isolates
548
281
86
metaG (82%)
isolates
464
195
57
metaG (66%
isolates
301
308
84
metaG (83%)
isolates
705
240
85
metaG (86%
isolates
606
222
66
metaG (77%
isolates
622
239
63
metaG (86%
isolates
496
308
89
metaG (89%)
isolates
534
342
112
metaG (80%)
isolates
597
310
84
metaG (84%)
isolates
411
381
154
metaG (84%)
isolates
